# Synergistic effect of MDM2 inhibitors and radiotherapy in endometrial cancer

**DOI:** 10.1038/s41698-025-01063-9

**Published:** 2025-08-18

**Authors:** Roberto Vargas, Aaron Petty, Daniel J. Lindner, Yvonne Parker, Brian Yard, Arda Durmaz, Kristi Lin-Rahardja, Ofer Reizes, Robert Debernardo, Jacob Scott

**Affiliations:** 1 Division of Gynecologic Oncology, Obstetrics and Gynecology Institute, The Cleveland Clinic, Cleveland, OH USA; 2 Department of Translational Hematology and Oncology Research, Lerner Research Institute, The Cleveland Clinic, Cleveland, OH USA; 3 Department of Cellular and Molecular Medicine, Lerner Research Institute, The Cleveland Clinic, Cleveland, OH USA; 4 Division of Radiation Oncology, Taussig Cancer Center, The Cleveland Clinic, Cleveland, OH USA

**Keywords:** Endometrial cancer, Radiotherapy, Targeted therapies

## Abstract

Endometrial cancer (EC) is the most common type of gynecologic malignancy in the United States, with over 69,120 new cases expected in 2025. The total number of mortalities surpasses that of ovarian cancer. Despite our ability to identify different biological clusters of EC, we have yet to understand the functional impact of key genomic alterations associated with varying prognoses and exploit this knowledge for therapeutic benefits. Our overarching goal is to understand how genomic alterations impact radiotherapy response in EC, and whether manipulation of these signaling pathways could be utilized as a radio-sensitization strategy. Given that *TP53-*mutated ECs portend the worst prognoses and seem to benefit from escalation of therapy above that of radiotherapy alone, we first focused our attention on understanding the impact of this genomic aberration on radiation response. Using high-throughput in vitro profiling, genomic manipulation, and in vivo studies, we demonstrated that p53 signaling plays a significant role in the radiotherapy response in EC, thus providing a biological rationale for observed clinical trial findings. We also leveraged this same finding to test a therapeutic approach driving p53/p21 signaling using murine double minute-2 (MDM2) inhibitors, subsequently demonstrating synergism with radiation. Thus, MDM2 inhibitors could be considered as a novel radiosensitizing approach for EC.

## Introduction

Endometrial cancer (EC) is the most common gynecologic malignancy in the United States, with over 69,120 new cases expected in 2025^1^. Current trends outpace prior projections, with a 55% increase in incidence predicted from 2010 to 2030^2^. Endometrial cancer mortality also surpasses that of ovarian cancer; it is the only malignancy for which survival rates have not improved in the last 30 years^3^. While there have been significant advances in identifying the genomic underpinnings of EC^4–8^ and their prognostic impact^9,10^, there remains a significant void in understanding the functional and therapeutic impact of the key genomic alterations found in EC, limiting our ability to exploit these alterations for therapeutic gain.

Historically, radiotherapy (RT) has played a key role in the prevention and treatment of both early-stage and advanced/recurrent EC^8,11–13^. The landmark GOG-258 trial challenged the value of radiotherapy in advanced/recurrent EC by demonstrating no improvement in progression-free survival (PFS) for chemoradiation over chemotherapy alone, but conferring increased toxicity in the chemoradiation arm^13^. Conversely, the PORTEC-3 study demonstrated that the addition of chemotherapy to radiotherapy did not improve PFS over RT alone, in the predefined study population^12^. It should be noted that the PORTEC-3 patient population included high-risk histologies but represents overall a lower risk population, based on the presence of early-stage patients. While these landmark studies provide alternative views on the value of chemotherapy and radiotherapy regarding PFS, they unanimously highlight the value of radiation for local disease control.

Subsequent analyses have since identified EC molecular subgroups that derive benefit from escalated therapy, including both concurrent chemoradiotherapy and systemic chemotherapy. In the PORTEC-3 population, patients with aberrant p53 staining (IHC) benefited from the addition of chemotherapy to the RT backbone, with an improved PFS regardless of histology^10^. In parallel, a molecular sub-analysis of GOG-258 found that patients with wild-type p53 staining in the chemoradiation arm had improved PFS over the group who received chemotherapy alone^14^. Both of these molecular sub-analyses support the notion that certain tumors derive less benefit from RT, based on genomic factors such as *TP53* status, but cannot provide direct biologic evidence to support specific resistance/sensitivity patterns. In fact there exist other prospective studies that challenge the mentioned paradigm in regards to *TP53* status and RT, demonstrating a clinical benefit to RT in those tumors harboring *TP53* alterations^15^. Given the conflicting clinical evidence, investigating the direct functional and therapeutic impact of p53 on the radiotherapy response in EC remains necessary. Understanding how different tumors may be more/less sensitive to RT alone may help provide the necessary biologic data to help design our clinical trial strata going forward.

Attempts to understand the functional impact of p53 on radiation response in disease sites remain nuanced, with some demonstrating no effect on radiation response in p53-aberrant malignancies^16,17^, and others noting a correlation with radiation resistance^18,19^. The tissue-specific effect of p53 signaling on the response to radiotherapy also remains unclear and inconsistent^20–22^. Given that 80% of endometrial cancers do not harbor *TP53* mutations, understanding the EC-specific impact of this pathway could create an opportunity to improve radiotherapy prescription on both sides of the mutational spectrum (wild type and/or aberrant). A plausible explanation could be the differential expression of the E3 ubiquitin ligase responsible for p53 degradation, also known as murine double minute-2 (MDM2). Inhibition of MDM2 by small-molecule biological agents, such as Nutlin-3, prevents the breakdown of wild-type p53 and promotes increased downstream apoptotic signaling^23–26^. A strategy for testing these small-molecule inhibitors remains unexplored in EC, likely due to the lack of consistent data regarding the impact of p53 signaling in EC and early clinical trials identifying significant hematologic toxicities with these inhibitors^27,28^. Despite the toxicity signals, the demonstrated efficacy and clinical feasibility of this approach are supported by an ongoing clinical trial in sarcomas, testing the role of a novel MDM2 inhibitor (AMG-232) as a radiosensitizer for soft-tissue sarcomas lacking *TP53* mutations (NCT03217266)^29^.

The absence of robust data regarding the functional and therapeutic impact of p53 status in EC has limited our ability to leverage these signaling pathways in a meaningful therapeutic manner. This drove our investigation to address the primordial question: Does *TP53* status impact radiotherapy response in EC? To directly assess the impact of *TP53* mutations on the radiotherapy response in EC, we integrated a high-throughput radiation phenotyping platform, intron-targeting CRISPR/Cas9 techniques, and dose-response assessment using cell lines. The renewed interest in leveraging this pathway for radio-sensitizing potential then prompted our investigation into MDM2 inhibitors. We performed in vitro and in vivo radiation sensitivity experiments with MDM2 inhibitors in cell lines and mouse xenograft models. Our findings confirm the functional impact of *TP53* status on RT response in endometrial cancer and encourage clinical exploration of MDM2 inhibitors as a novel therapeutic approach in EC.

## Results

### *TP53* status impacts radiotherapy response in EC cell lines

**Fig. 1:**
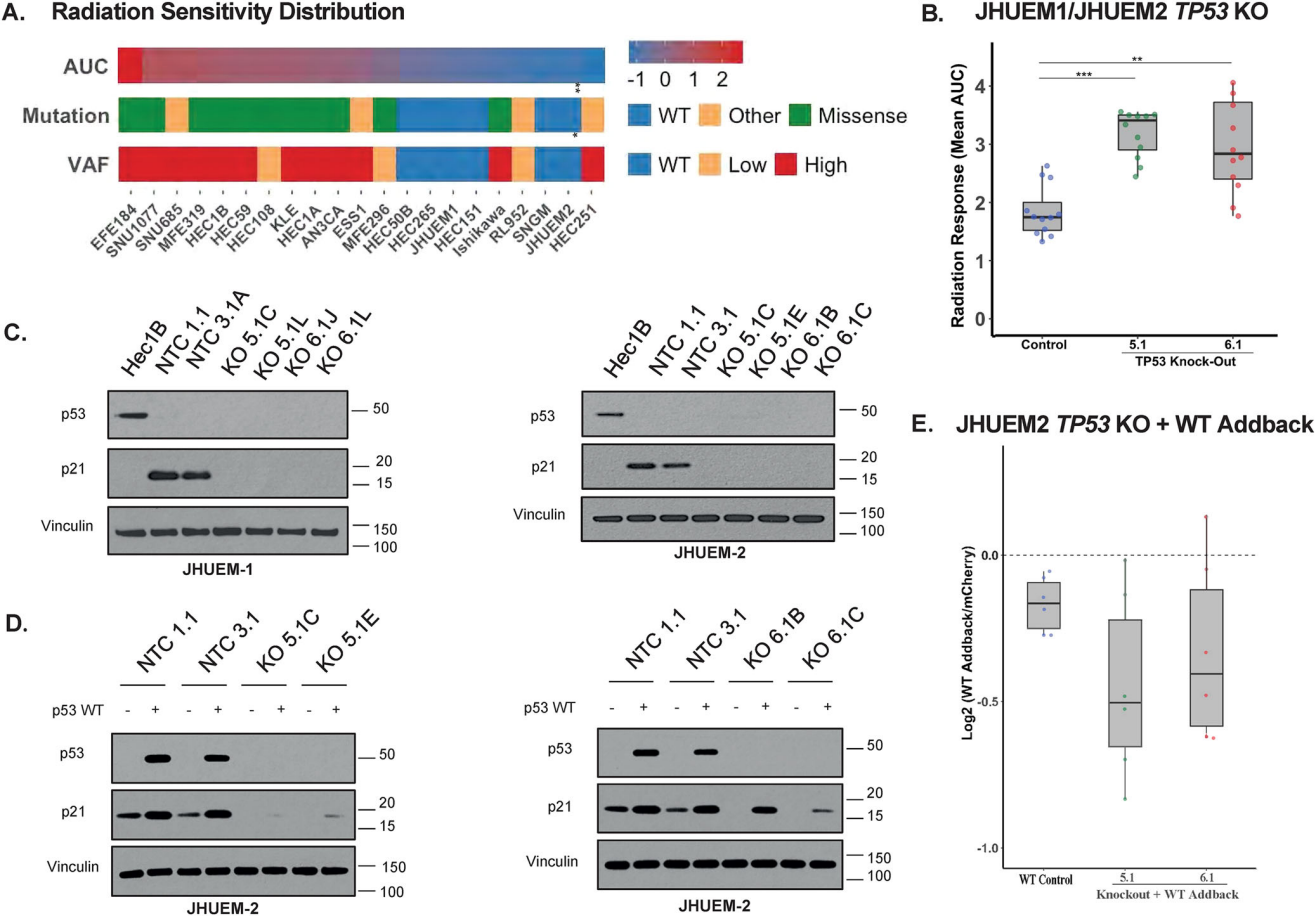
*TP53* status and allelic frequency are associated with radiation resistance in EC cell lines. **A** Heat-map depicting 21 EC cell lines organized by decreasing radiation resistance (left to right) as quantified by area under the curve (AUC). The cell lines’ *TP53* mutational status and variant allelic frequency (VAF) are depicted in the bars below. A significant association between AUC and *TP53* mutation status (*p* = 0.004) or VAF (*p* = 0.01), respectively. A correlation between mutation and AUC (LR = 11.12 and *p* = 0.0038) as well as VAF and AUC (LR = 9.42 and *p* = 0.009) was observed. **B** The mean AUC for JHUEM1 and JHUEM2 cell lines are organized based on the CRISPR/Cas9 guide-RNA (gRNA) sequence for *TP53* KO, as compared to the non-targeting control (NTC). Kruskal–Wallis test with Dunn’s Multiple Comparisons Test was performed with *p* < 0.001 for NTC vs KO 5.1, *p* = 0.0016 for NTC vs KO 6.1. **C** Western-blot analysis of two monoclonal isolates from each gRNA and NTC reveals loss of p21 signaling with knock-out of *TP53* in JHUEM1 (left) and JHUEM2 (right). **D** Western blot analysis for downstream signaling after wild-type *TP53* addback to 2 different KO clones of JHUEM2 under two different gRNAs (5.1 left / 6.1 - right). **E** Box-whisker plot depicting the Log2 fold-change in radiation response AUC with wild-type *TP53* addback to JHUEM2 knock-out clones. Dunnett’s Multiple Comparisons test was performed with *p* = 0.0051 for KO 5.1 C vs NTC, *p* = 0.0199 for KO 6.1 C vs NTC.

To examine the association of *TP53* mutations and their allelic frequencies with radiation resistance, we correlated *TP53* status with gamma radiation response in EC cell lines, using area under the curve (AUC) measures from a previous pan-cancer profiling effort^30^. Endometrial cancer cell lines with an altered *TP53* status were more likely to have higher AUC and thus increased radiation resistance (LR 11.12, *p* = 0.004). Given the potential impact of allelic frequency on the therapeutic response, we also annotated the analysis based on allelic frequencies reported in the Cancer Cell Line Encyclopedia^31^. When stratified by allelic frequency, those with a VAF > 0.5 (high VAF) were also more likely to be associated with radiation resistance/higher AUC (LR 9.42, *p* = 0.009), as seen in Fig. 1A. These findings support the hypothesis that heterozygous *TP53* missense mutations act as dominant-negative alleles, allowing for only a moderate decrease in radiation sensitivity due to the wild-type allele that remains present.

Using an intron-targeting CRISPR/Cas9 system allowed us to knock out (KO) *TP53* and subsequently add back clinically-relevant gene variants. A statistically significant increase in the AUC (representative of radiation resistance) was observed in JHUEM1 and JHUEM2 cell lines (both wild-type *TP53)* after CRISPR/Cas9 KO of *TP53* (Fig. 1B). As noted by western blotting, signaling through the p53/p21 pathway was disrupted in KO cell lines (Fig. 1C). No discernible change in the AUC for the radiation response was observed in CRISPR/Cas9 *TP53* knockouts in the Hec1A, Hec1B, and MFE-319 cell lines, all of which contained high-VAF GOF/DN *TP53* alterations (Supplementary Fig. 1).

Complementation with a low-expression (PGK) vector containing wild-type *TP53* re-established p53/p21 signaling (Fig. 1D) and radiation sensitivity (Fig. 1E) in JHUEM1/JHUEM2 *TP53* knockouts *(TP53*-KO*)*. Of note, complementation with wild-type TP53 in TP53-KO versions of copy-number-high cell lines that originally harbored variant alleles (Hec1A, Hec1B, and MFE319) did not reintroduce significant radiation sensitivity. It is plausible that the evolutionary trajectories of these *TP53-*mutant cell lines have allowed for the amplification of other established genomic drivers of resistance, as evidenced by their increased somatic copy number alterations, in addition to the direct impact on p53/p21 signaling.

### Gain-of-function/dominant-negative *TP53* mutations negatively impact radiotherapy response

**Fig. 2:**
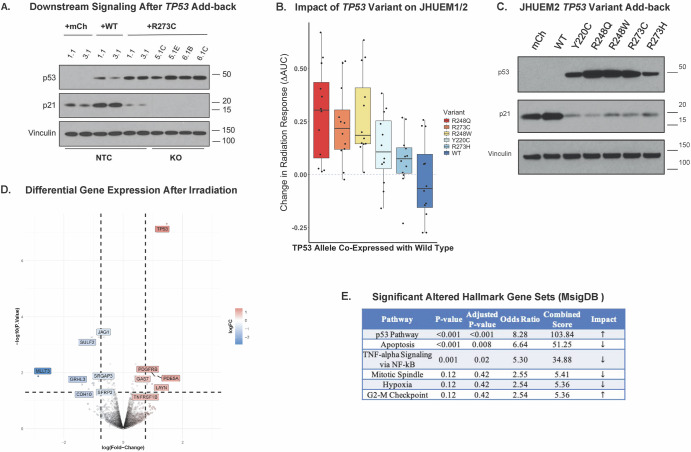
Gain-of-function/dominant-negative alleles of *TP53* impact p21 signaling, radiation response, and apoptotic pathways. **A** Western-blot analysis of JHUEM2 non-targeting control [NTC] isolates (1.1 and 3.1) with the addition of mCherry (complementation transfection control), wild-type *TP53* (over-expression), and the addition of the R273C allele (co-expression with wild-type). **B** Waterfall plot depicting the overall effect on AUC (Log2-fold change) after introduction of a variant allele (or wild-type) across four different mono-clonal NTC isolates, NTC 5.1 and 6.1 for both JHUEM1 and JHUEM2. **C** Western-blot analysis of JHUEM2 NTC 3.1 (wild-type control) after complementation with each GOF/DN *TP53* variant. **D**, **E** JHUEM2 isolate with R273C co-expression and the native NTC (wild-type alleles only) were irradiated at 2 Gy. RNA extracted 24 h after irradiation and gene expression assessed via RNAseq. On the volcano plot, the x-axis denotes a 1.5-fold change (FC) on a log2 scale and *y*-axis denotes a 0.05 false discovery rate (FDR) on a -log10 scale (**D**). Hallmark gene set analysis performed using MsigDB (**E**).

Employing the same *TP53-KO* constructs, we subsequently explored the impact of five hotspot *TP53* alterations (R248Q, R248W, R273C, R273H, and Y220C) on RT response^32,33^. First, the effect of each mutation on p53/p21 signaling and radiation phenotype was assessed. We noted that the introduction of these hotspot variants into JHUEM2 *TP53-*KO (5.1, 6.1) cell lines did not re-establish p53/p21 signaling (Fig. 2A, Supplementary Fig. 2) or alter the radiation response. When the mutations were co-expressed with wild-type *TP53* present in the non-targeting controls (NTC 1.1 and 3.1) for JHUEM1 and JHUEM2, a dominant-negative effect was observed. These NTC clones were infected with a random guide RNA as our CRISPR control and thus retain their parental wild-type alleles. The expression of five *TP53* hotspot variants led to an increase in radiation resistance despite the presence of the two parental wild-type alleles (Fig. 2B) and concurrently, abrogation of p53/p21 signaling (Fig. 2C). These findings support the hypothesis that p53 signaling through the p21 pathway is negatively affected by these variants and contributes to resistance via a dominant-negative mechanism.

To explore the potential off-target effects of dominant-negative variants, we exposed our JHUEM2 non-targeting control, with and without co-expression of the R273C allele, to 2 Gy of gamma radiation. RNA was extracted 24 h later and differential gene expression analysis was performed. The hallmark pathways that were significantly altered in two replicates of *TP53* R273C co-expressing cell lines (1.1RC and 3.1RC) included apoptosis and tumor necrosis-alpha gene sets, mainly driven by the downregulation of *CDKN1A, BTG2, CCND1, GADD45B, NINJ1, IER2, and FAS* (Fig. 2D, E, Supplementary Fig. 3, Supplemental Table 1).

### MDM2 inhibitors impact cell viability and radiotherapy response via increased p53/p21 signaling

**Fig. 3:**
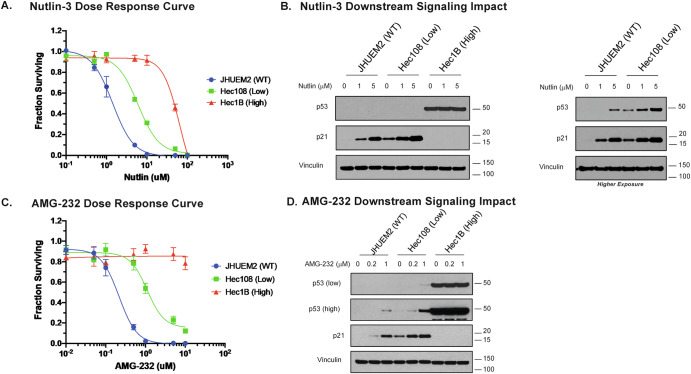
MDM2 inhibition with nutlin-3 and AMG-232 as a cytotoxic strategy. **A**, **C** Drug response curve for treatment of EC cell lines representing three different allelic frequency populations – JHUEM2 (wild-type), Hec108 (heterogeneous-low VAF), and Hec1B (homogenous – high VAF) with increasing doses of Nutlin-3 (**A**) and AMG-232 (**C**). Error bars indicate standard error of the mean (SEM). Both demonstrate significant effects on cell viability in JHUEM2 and Hec108. Hec1B was resistant to treatment with either drug. **B**, **D** Western-blot analysis of all three cell lines after treatment with increasing doses of either Nutlin-3 (**B**) or AMG-232 (**D**), demonstrating an increase in p53/p21 with increasing concentrations of either drug.

Given the demonstrated significance of the p53/p21 signaling pathway in the EC cell line radiotherapy response, we tested the hypothesis that MDM2 inhibitors could serve as cytotoxic and/or radiosensitizing agents in EC. We tested both a first-generation MDM2 inhibitor, Nutlin-3, and a novel MDM2 inhibitor, AMG-232. The latter is currently undergoing clinical investigation in soft-tissue sarcomas alongside RT. Using three parental cell lines to replicate wild-type (JHUEM2), heterozygous *TP53*-mutated (Hec108), and homozygous *TP53-*mutated (Hec1B) EC cases, we performed cell viability experiments using both Nutlin-3 and AMG-232. A differential effect on cell viability was observed depending on the presence of wild-type *TP53* and the allelic frequency of the mutant *TP53*. Only the cell lines with a wild-type allele (JHUEM2 and Hec108) showed decreased cell viability (Fig. 3A) and downstream p21 activation (Fig. 3B) in response to treatment with Nutlin-3. Treatment of JHUEM2 and HEC108 with AMG-232 had a more potent impact on cell viability (Fig. 3C) and induction of p21 (Fig. 3D) when compared with Nutlin-3. Hec1B, homozygous for the *TP53* R248Q mutation, was resistant to treatment with both MDM2 inhibitors, which is consistent with previous studies^34^.

**Fig. 4:**
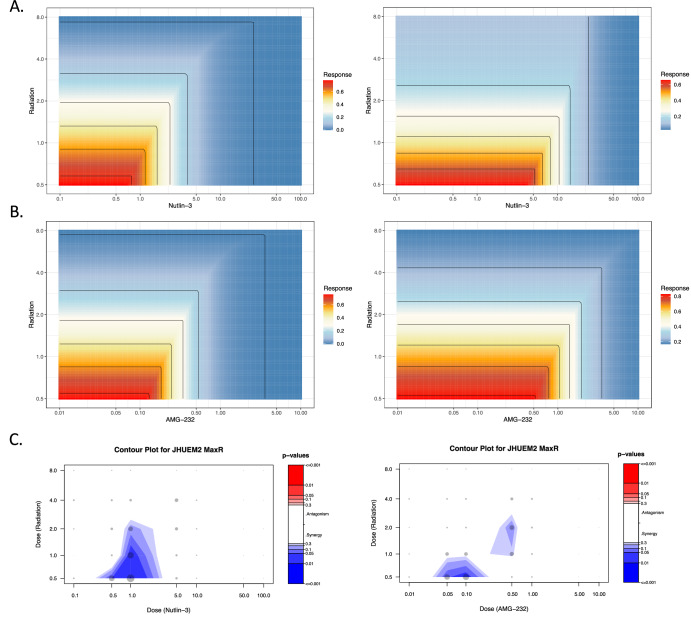
MDM2 inhibitors are synergistic with radiotherapy in vitro*.* Isobolograms for Nutlin-3 (**A**) and AMG-232 (**B**) in two EC cell lines - JHUEM2 (left) and Hec108 (right) after gamma irradiation. Drug dosage is the x-axis and radiation dose is the *y*-axis. **C** Contour plots depicting areas of calculated synergy (purple) between radiation and Nutlin-3 in JHUEM2 (left), as well as AMG-232 in JHUEM2 (right). Synergy was quantified by comparing actual values to those predicted by fitting the Highest Single Agent (HSA) model to the data.

We then tested the effect of MDM2 inhibition on radiation response using Nutlin-3 and AMG-232. When cell lines were treated with Nutlin-3 and exposed to a range of doses of gamma radiation, a synergistic effect was observed in JHUEM2 and an additive effect in Hec108 (Fig. 4A, C). Treatment with AMG-232 demonstrated a synergistic effect in both JHUEM2 and HEC 108 cell lines (Fig. 4B, C, Supplementary Fig. 4).

### AMG-232 is synergistic with radiotherapy in a xenograft model of wild-type *TP53* endometrial cancer

**Fig. 5:**
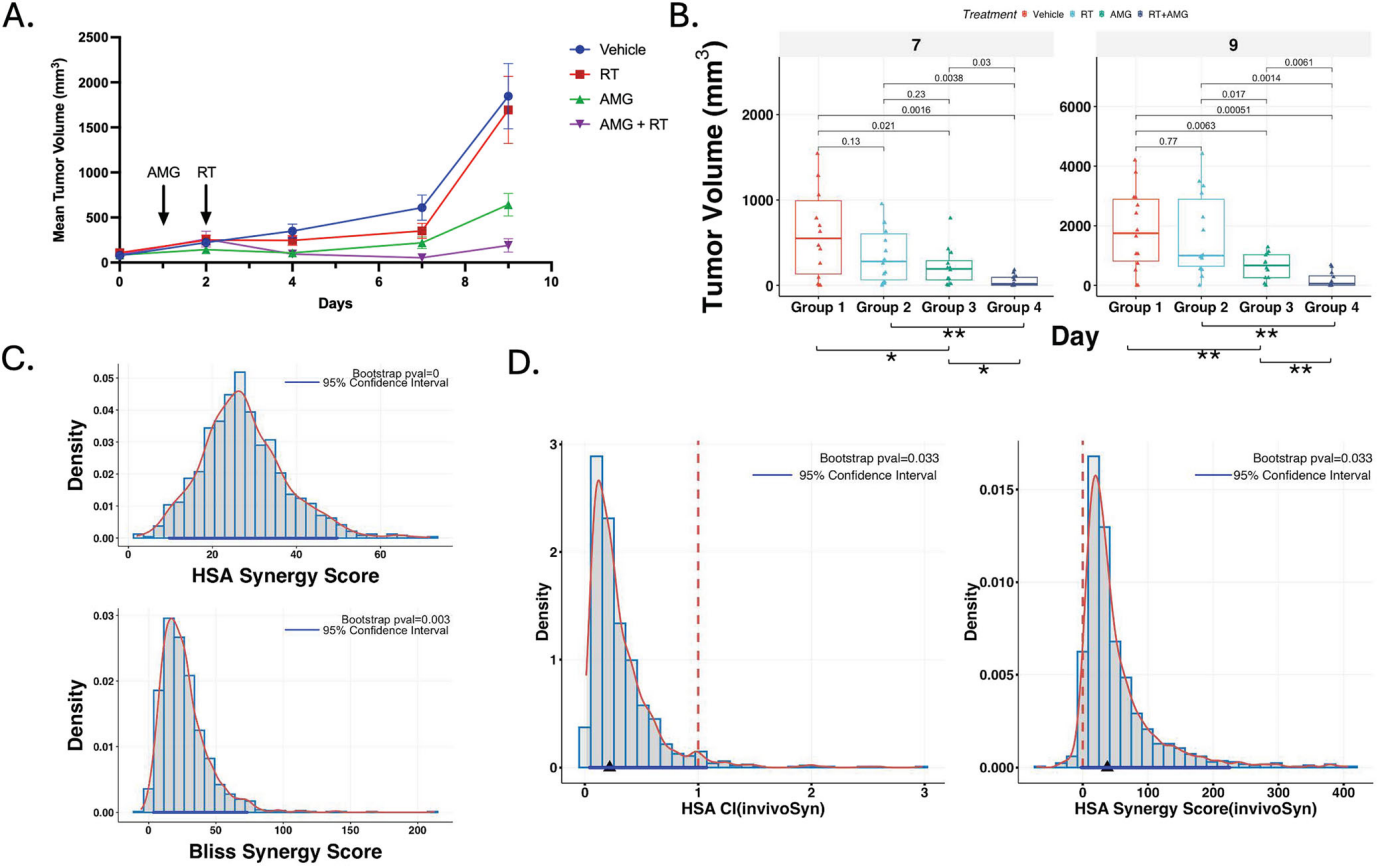
In vivo synergistic effect of AMG-232 and radiotherapy. **A** The tumor growth curves demonstrate the mean tumor volume over time of each treatment group - vehicle, AMG-232, 1 Gy radiotherapy (RT), combination AMG-232 and RT. Five mice per group, each with bilateral flank tumors were assessed. Error bars indicate standard error of the mean (SEM). Arrows indicate both the start of AMG-232 treatment and single RT dose. **B** Box-whisker plots demonstrating the median tumor volume at days 7 and 9 of treatment. *P*-values for group comparisons were calculated using pairwise *t*-test, with *p* < 0.05 indicating significance. A statistically significant difference between the AMG-232 containing treatment groups and vehicle/RT alone groups is observed. Notably, the combination therapy group was also significantly smaller when compared to the AMG-232 alone group. **C** Density plots of calculated synergy scores with bootstrap analysis of % tumor growth inhibition (TGI) values at day 9 of treatment using HSA and Bliss models. Synergy score was calculated as observed combo TGI – expected combo TGI, with synergy score >0 indicating synergy. **D** Density plots of calculated synergy scores and combination index values (CI) from bootstrap synergy analysis of the entire growth curve, as captured by AUC, using HSA model, This data demonstrates synergy under the HSA model with a combination index (CI) of <1 and synergy score >0.

To assess the in vivo efficacy of this therapeutic approach, we generated xenograft models of the JHUEM2 cell line and assessed the response to radiotherapy with and without AMG-232. Given the relative sensitivity of the JHUEM2 cell line in in vitro experiments, we employed a 1 Gy single fraction of irradiation. A single fraction of radiotherapy (RT) did not significantly alter tumor growth, whereas AMG-232 as a single agent and the combination of RT with AMG-232 both slowed tumor growth (Fig. 5A). At day 7 and 9 post-RT, a significant difference was observed between both AMG-232 treatment groups (+/− RT) when compared to the RT or vehicle group. (Fig. 5B, Supplemental Fig. 5) There was also a significant difference between the combination group when compared to single agent AMG-232. Subsequently, we performed two synergy analyses using tumor growth inhibition (TGI) at day 9 and the growth curve (represented as AUC) for comparison. Using the highest single agent (HSA) and Bliss independence models, a statistically significant synergy was observed on day 9 post-RT under both conditions (Fig. 5C, Supplementary Fig. 6). Considering the entire curve using an AUC measure, the HSA model retained statistically significant synergy (Fig. 5D), while the more restrictive Bliss model fell outside of statistical significance (not shown).

## Discussion

Our integrative analysis incorporating high-throughput radiation phenotyping, complementation experiments using CRISPR/Cas9, and pharmacologic targeting provides a fundamental biological rationale behind the disparate clinical outcomes in EC and supports further preclinical investigations. Although the prognostic value of *TP53* status in EC has previously been observed in clinical trials, the precise therapeutic impact remains poorly understood. Using cell lines, we demonstrated the oncogenic gain-of-function impact and dominant-negative effects of *TP53* variants in EC. We also established that not all *TP53*-mutated cell lines were resistant to MDM2 inhibition, and the degree of resistance was dependent on the amount of functional wild-type p53 present. Lastly, we demonstrated the potential for radio sensitization using MDM2 inhibitors in in vitro and in vivo models of EC.

Given that the majority (80%) of EC has wild-type *TP53*, we first focused on wild-type or low-grade cell lines to demonstrate that the loss of this key tumor suppressor results in the development of radiation resistance. Subsequently, we noted that restoration of the wild-type allele re-established downstream WAF/p21 signaling and restored a small degree of radiation sensitivity. We attribute the lack of a complete restoration in the radiation response phenotype to the low expression of wild-type *TP53* by the PGK promoter in our vector. The same complementation approach was used to assess common hotspot mutations (Y220C, R273C/H, and R248Q/W) and their functional impacts. While the mutations themselves provided a GOF phenotype by conferring radioresistance, they also exhibited a dominant-negative effect on wild-type signaling. Despite being a low-expression vector, the introduction of all five missense mutations suppressed p21 signaling established by the two parental wild-type *TP53* alleles. Furthermore, we demonstrated that GOF/dominant-negative alleles also confer a radioresistant phenotype.

Gene expression changes observed after treatment with 2 Gy of radiation support the hypothesis that these GOF mutations may target other pathways relevant to the radiation response. Our pathway analysis showed that genes associated with apoptotic pathways and tumor necrosis factor-alpha (TNF-α) signaling via NF-κB were downregulated in the presence of the R273C allele and irradiation. Pal et al. previously demonstrated that TNF-α influences radiation-mediated DNA damage, with decreased TNF-α leading to reduced double-stranded breaks in lung cancer cell lines^35^. Additionally, the same authors found that serum TNF-α expression was positively correlated with clinical outcomes after radiotherapy. The degree of TNF-α induction after irradiation has also been shown to predict the clinical radiotherapy response in multiple solid tumors^36^. While TNF-α/NF-κB signaling has been shown to have both pro-apoptotic and anti-apoptotic properties based on cell/tumor lineage^37^, in our *TP53-*altered (R273C) EC cell line, we observed a decrease in the expression of genes associated with TNF-α/NF-κB signaling, as well as other apoptosis-related genes when treated with radiation. Previous studies in prostate cancer cell lines have also noted that a decrease in NF-κB is associated with anti-apoptotic effects after radiation^38,39^. Crosstalk between p53 and TNF-α/NF-κB through non-canonical pathways may contribute to cell survival after irradiation^40^.

This ongoing need for novel treatment strategies incorporating radiotherapy in EC has been recognized by the NRG Oncology, which sponsored a randomized trial (GOG-0238) to address this specific clinical need^41^. We assessed the potential impact of pharmacological exploitation of intact/wild-type p53 signaling using two different MDM2 inhibitors. Both Nutlin-3 and AMG-232 increased p53/p21 signaling and conferred a synergistic effect on parental cells with wild-type *TP53*, particularly at low doses of radiation and MDM2 inhibitors. Furthermore, we noted that even in the presence of a GOF mutation, MDM2 inhibitors provide an additive effect when administered in combination with radiotherapy, presumably by leveraging the remaining wild-type copy in heterozygous mutations. This further broadens the potential application of MDM2 inhibition in EC by including some tumors with altered, but not absent, p53 signaling. The in vivo experiments further support the exploration of MDM2 inhibitors to improve the response of EC to radiotherapy. Although radiotherapy dosing in our experiment was lower than clinical dosing (1 Gy versus standard 1.8 Gy clinical dose) and administered as a single fraction, significant synergy between radiotherapy and AMG-232 was observed. The improved tumor response at clinically relevant doses introduces the rationale to further study these agents to bolster response to RT or reduce the amount of radiotherapy needed to achieve a complete response.

In summary, our work provides the much-needed biological rationale for the observed clinical outcomes of many gynecologic oncology trials; *TP53* alterations confer resistance to radiotherapy in EC. Furthermore, we demonstrate that dominant-negative alleles affect the radiation response via abrogation of the canonical p53/p21 pathway and may also interfere with the TNF-α response. Lastly, we provide in vitro and in vivo data supporting the investigation of MDM2 inhibitors as a novel radiosensitizing agent in this disease site, with the potential to broaden its impact by including those with heterozygous *TP53* mutations. Taken together, our data support established clinical trials in EC and advocate for genomics-driven clinical trials that incorporate biologics with radiotherapy in recurrent and early-stage EC.

## Methods

### Cell culture

JHUEM1, JHUEM2, and Hec108 cells were grown in DMEM-F12 medium containing 15 mM HEPES and 2.5 mM L-Glutamine (Thermo Fisher, MA, USA) supplemented with 10% FBS, 100 U/mL Penicillin, and 100 μg/mL streptomycin. The Hec1A, Hec1B, MFE319, and HEK293T cell lines were grown in Dulbecco’s modified DMEM media containing 4.5 g/L Glucose, 1 mM Sodium Pyruvate, 4 mM L-glutamine (Thermo Fisher, MA) supplemented with 10% FBS, 100 U/mL Penicillin, and 100 μg/mL streptomycin. All the cultures were maintained at 37 °C in a humidified atmosphere containing 5% CO_2_. All cell lines were verified by human short-terminal repeat (STR) analysis (Labcorp, NC, USA) and tested to ensure the absence of *Mycoplasma* via qPCR.

### Lentivirus production and infection

Lentiviruses containing all plasmids were generated by transfecting HEK293T cells with the appropriate plasmid along with psPAX2 (Addgene; Plasmid #12260) and pMD2.G (Addgene; Plasmid #12259) plasmids in a 5:4:1 ratio. Approximately 18 h after transfection, the medium was changed, followed by collection and purification of lentiviral-containing supernatants over the next two days. Centrifugation and passage through a 40 µM filter were used for purification. Cell lines were infected with these viruses at an MOI > 0.8 in media supplemented with 5 ug/ml polybrene.

### Exon-intron junctional CRISPR

sgRNAs targeting the exon 5-intron (5.1) and exon 6-intron (6.1) junctions of *TP53* were designed using the Synthego CRISPR web design tool (https://www.synthego.com/products/bioinformatics/crispr-design-tool/). Non-targeting control (NTC) sgRNAs were also designed. For each sgRNA, two complementary oligos with BsmBI-digested overhang sequences were designed. The sgRNA sequences are shown in Supplementary Table 2. plentiCRISPRv2-Blast plasmid (Addgene: Plasmid #83480), which contains the Cas9 coding sequence and a cloning site for sgRNA, was digested with BsmBI, followed by annealing of sgRNAs and ligation into the pLentiCRISPRv2-Blast plasmid, as detailed on the website for the original pLentiCRISPRv2 plasmid (Addgene #52961)^42,43^. Plasmids were transformed into NEB Stable bacteria (New England Biolabs, MA) and colonies were screened for sgRNA inserts by Sanger sequencing of the ligation site. After viral infection, the cells were selected and maintained in the presence of 5 μg/mL blasticidin (Thermo Fisher, MA). Monoclonal cells were isolated from a polyclonal pool of transduced stable cells using a limiting-dilution assay. Protein lysates were obtained from each clone, and *TP53* KO was confirmed using western blotting.

### Variant-expressing plasmid generation

Variant-expressing lentiviral plasmids were generated by PCR-based site-directed mutagenesis, followed by bacterial recombination and transformation. First, 5′ and 3′ fragments containing the mutated gene open reading frame (ORF) sequences were amplified by PCR from the pDONR223-*TP53* WT plasmid (Addgene; Plasmid #82754). The 5′ and 3′ flanking PCR primers were located at the attL1 and attL2 sites, with the internal primers containing the mutated sequences. The sequences of the PCR primers are shown in Supplementary Table 3. Fragments were then transferred to the destination vector pLEX306 (Addgene; Plasmid #49391) by LR reaction with LR Clonase II enzyme (Invitrogen, CA, USA), followed by transformation into competent cells. The overlapping fragment overhangs at the mutation site were repaired by endogenous bacterial repair. The inserted sequences and incorporated mutations were verified by restriction digestion with BsrGI and Sanger sequencing of the ORF, respectively.

### Complementation

Wild-type and variant containing ORFs of *TP53* were cloned into pLEX306 and integrated into CRISPR KO or non-target control (NTC) cell lines. After viral infection, the cells were selected and maintained in the presence of 1 μg/mL puromycin.

### Antibodies and reagents

Anti-p53 (#9282, 1:3000), anti-p21 Waf1/Cip1 (clone 12D1, #2947, 1:3000), and anti-vinculin (clone E1E9V, #13901, 1:5000) antibodies were purchased from Cell Signaling Technology. Goat anti-rabbit antibody linked to horseradish peroxidase (HRP) was used as a secondary antibody (Santa Cruz Biotechnology, CA, USA).

### Western blot analysis

Cells were pretreated with 10 uM Nutlin-3 in 0.1% DMSO for 18 h to enhance the protein signal to a detectable level for comparison. Cells for dose-response analyses were treated with an appropriate dose of 0.05% DMSO. Whole-cell lysates were prepared using M-PER lysis buffer (Thermo Fisher, MA, USA) and clarified by centrifugation. Proteins were separated by SDS–PAGE on 4–12% Bis-Tris gradient gels (Thermo Fisher, MA) and transferred onto 0.2 μM nitrocellulose membranes (Bio-Rad, CA). Membranes were blocked and incubated with the primary antibody for 2 h at room temperature, followed by washing and incubation for 1 h with a secondary antibody. Blots were developed using an ECL Plus chemiluminescence system (Amersham, GE Healthcare, UK).

### High-content radiation assay

Cells were plated using a Multidrop Combi liquid handler (Thermo Fisher, MA) at multiple cell densities in white 96-well plates (Corning, NY) for each cell line. Cells were plated in six replicate wells for each cell density in triplicate. Plates were irradiated, and 9 days post-irradiation, media were aspirated and 50 μL CellTiter-Glo^®^ reagent (50% solution in PBS) (Promega, WI) was added to each well. Relative luminescence units (RLU), proportional to the amount of ATP present, were measured using a Synergy H1 luminescence plate reader (BioTek, IL, USA). The luminescence signal was plotted as a function of cell density, and the cell density within the linear range for luminescence was selected to generate the integral survival for each cell line^30^. Cells were treated with γ-radiation delivered at 0.92 Gy/min with a ^137^Cs source using the Mark 1 Irradiator (Shepherd and Associates, CA). The area under the curve (AUC) was estimated using a trapezoidal approximation in GraphPad Prism software (Version 8.4.3, GraphPad, CA, USA). Radiation doses of 0, 2, 4, 6, and 8 Gy were administered and the surviving fraction was determined by normalizing each RLU value to the 0 Gy value. These data were used to generate an AUC value for each cell line.

### Drug dose-response assays

For drug dose-response experiments, cells were plated as described above and treated with either 0.5% DMSO (control) or seven different doses of the drug in 0.5% DMSO. For Nutlin-3, doses of 0.1, 0.5, 1, 5, 10, 50, 100 uM were used and 0.01, 0.05, 0.1, 0.5, 1, 5, 10 uM for AMG-232. Cells were treated for 24 h, followed by replacement with drug-free medium and incubation for 9 days. Cells were then lysed with CellTiter-Glo reagent and RLU values were obtained as described above. Fraction survival was determined by normalizing each RLU value to either the 0 Gy value, or the maximum value, whichever was greater. Four-parameter sigmoid curves were fitted to the dose-response data, and IC50 values were determined using GraphPad Prism software (Version 8.4.3, GraphPad, CA, USA).

### Drug radiosensitization assays

For drug radiosensitization experiments, cells were plated and treated with different drug doses, as described above. Following replacement with drug-free medium, radiation doses of 0, 0.5, 1, 2, 4, and 8 Gy were administered, followed by incubation for 9 days. For JHUEM2 and Hec108, cells in plates receiving 2, 4, and 8 Gy of radiation were plated at twice the density to allow for accurate RLU signal detection and comparisons. Fraction survival was determined by normalizing each RLU value to the untreated (0 µM, 0 Gy) values. The fraction surviving values at 2, 4, and 8 Gy were halved to correct for higher plating densities. The mean values for three replicates were obtained and all were divided by the maximum signal. These data were used to perform a synergy analysis.

### Xenograft radiosensitization experiments

Animal experiments using mice were performed in accordance with the recommendations of the Guide for the Care and Use of Laboratory Animals of the National Institutes of Health and conducted under a protocol approved by the Cleveland Clinic Institutional Animal Care and Use Committee (IACUC). Tumor inoculations were performed heterotopically and subcutaneously in both flanks of 6–8-week-old female *nod-*SCID gamma (NSG) mice (*n* = 20) using the JHUEM2 cell line. Five mice were assigned to each treatment group (vehicle, radiation, AMG-232, or combination therapy). Treatment was initiated when the largest tumor in each mouse reached approximately 200–300 mm^3^. Mice received vehicle gavage or AMG-232 daily, depending on the treatment group. Gavage treatment commenced 24 h prior to irradiation. Mice in the radiation and combination therapy groups were subjected to a single fraction of 1 Gy gamma radiation using a Shepherd irradiator. Tumors were measured thrice per week using calipers in two perpendicular dimensions. Tumor volume was calculated using the formula for a prolate spheroid: v = 4/3 × π × a^2^ × b, where a is the minor radius and b is the major radius.

### RNA Isolation and RNA Seq

For RNA isolation from cell lines, cells were first lifted off plates, washed with 1X PBS, and pelleted by centrifugation. These pellets were then lysed and RNA was extracted using the AllPrep DNA/RNA Mini Kit (Qiagen, MD). RNA Seq analysis was performed using the in-house Genomics Core Facility at the Cleveland Clinic. Briefly, the total RNA samples were enriched for mRNAs, followed by library preparation and fragmentation using an Illumina TruSeq Kit (Illumina, CA, USA). Paired-end 100 bp sequencing reads were generated using an Illumina NovaSeq 6000 machine (Illumina, CA, USA). RNA-Seq read pre-processing was performed using *fastp* with a low-complexity filter set and trimmed from both the 3’ and 5’ ends^44^. Reads were then aligned to the human genome gencode v40 using STAR aligner in gene quantification mode with 2 pass enabled^45^. Differential expression analysis was performed using the R package *edgeR*^46^. Pathway enrichment was completed using the Hallmark Gene Set from MsigDB, and *p*-values were calculated via hypergeometric distribution^47^.

### Graphs and statistical analyses

Statistical analyses for cell line data were performed using GraphPad Prism software (Version 8.4.3, GraphPad, CA). Graphs were generated using both GraphPad Prism software and R statistical analysis software. R code for graphs, tables, and statistical analyses are available on GitHub at the following address (github.com/The-Vargas-Lab/TP53-and-Radioresistance). The BIGL package was used to perform synergy analyses using the Highest Single Agent (HSA) additivity model for the JHUEM2 and Hec108 cell lines^48^. The Loewe additivity model was used for Hec1B as data did not meet the requirements for HSA. The data was then compared, and both magnitude and significance of deviation of each point from the model were calculated. For in vivo experiments, the *invivosyn* package for R software was used to generate graphs and perform synergy analyses. Both HSA and Bliss models were employed for comparison^49^.

## Supplementary information

Supplementary information

## Data Availability

RNA sequencing data will be publicly available through Sequence Read Archive (SRA) at time of publication https://github.com/The-Vargas-Lab/TP53-and-Radioresistance.

## Code availability

All R-programming code used in this manuscript is available in GitHub via https://github.com/The-Vargas-Lab/TP53-and-Radioresistance
