# Supplementary material for: Synergistic effect of MDM2 inhibitors and radiotherapy in endometrial cancer
Source: NPJ Precis Oncol. 2025 Aug 18;9:290. doi: 10.1038/s41698-025-01063-9 (PMC12361404; doi:10.1038/s41698-025-01063-9)

Supplemental Figure 1.

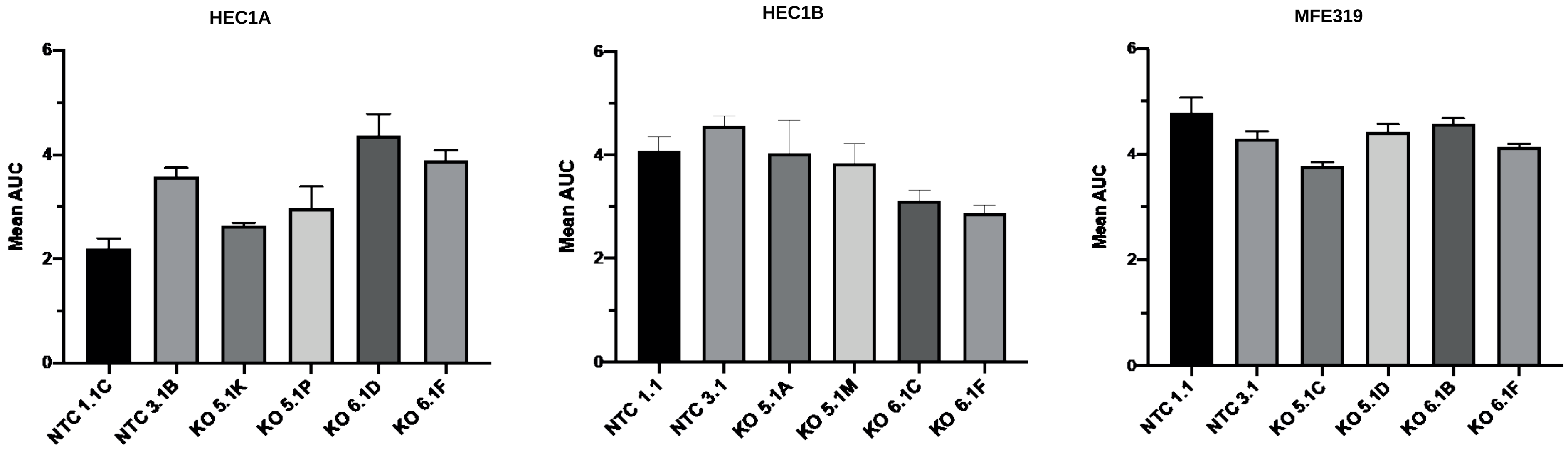

TP53 knockout via CRISPR/Cas9 in three EC cell lines with GOF/DN alleles and high VAF (presumed loss-of-heterozygosity) does not impact radiation response.

Supplemental Figure 2.

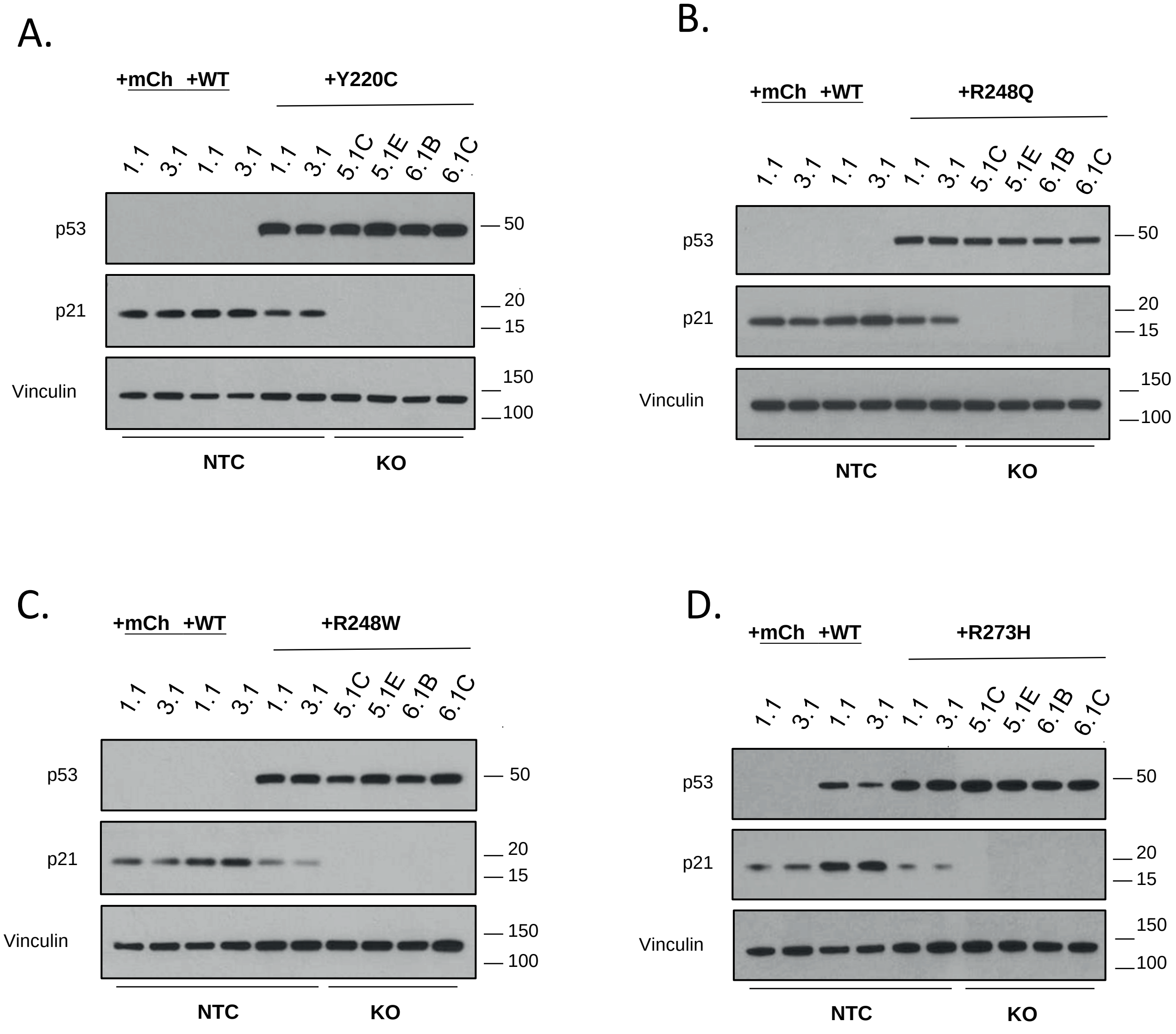

Western-blot analysis of 4 alternative TP53 alleles reveal abrogation of p21 signaling by the mutant allele in the presence of two wild-type alleles. As seen in the knock-out monoclonal lines (5.1C/E and 6.1B/C) expression of the mutant allele alone does not impact p21 signaling but confers significant accumulation of p53. Wild-type p53 is rapidly degreaded by MDM2, thus there is variability in the western blot ability to pick up the wild-type p53 band (in the 1.1/3.1 mcherry and +WT), as seen in panel D versus A-C.

Supplemental Figure 3.

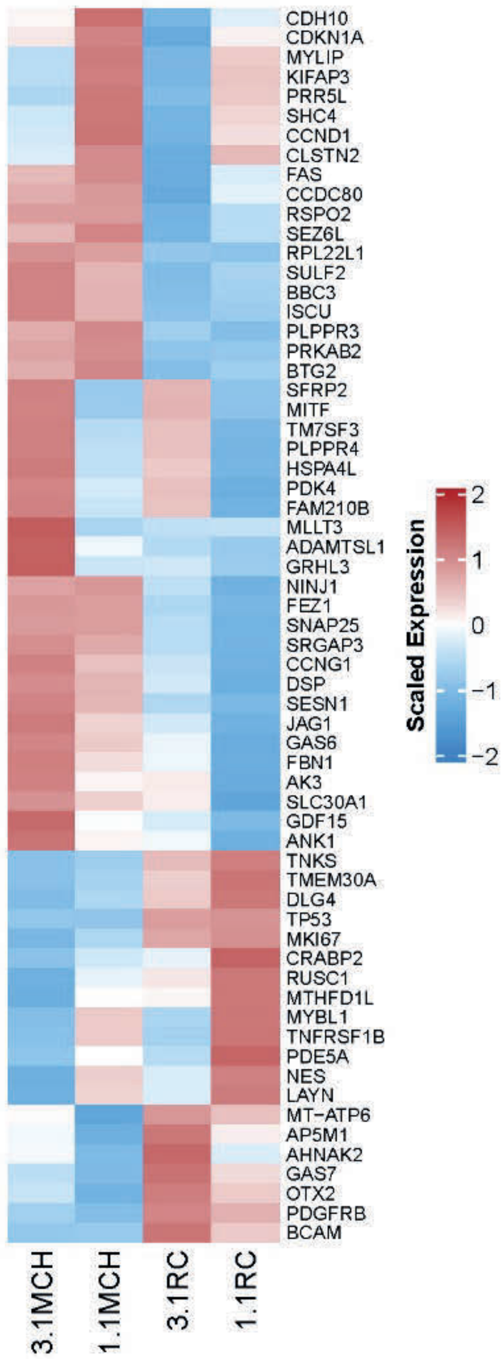

Heat-map of differential gene expression. In this experiment, two NTC (1.1 and 3.1) monoclonal isolates were transfected with mcherry and an R273C allele. Radiation was administered (2Gy) and RNA extracted 24 hours later. Differential gene expression between the lines with only wild-type alleles and those with wild-type + R273C can be appreciated. Notably TP53 expression is confirmed in our 1.1RC and 3.1RC constructs, confirming increased expression via our PGK promoter.

Supplemental Figure 4.

A

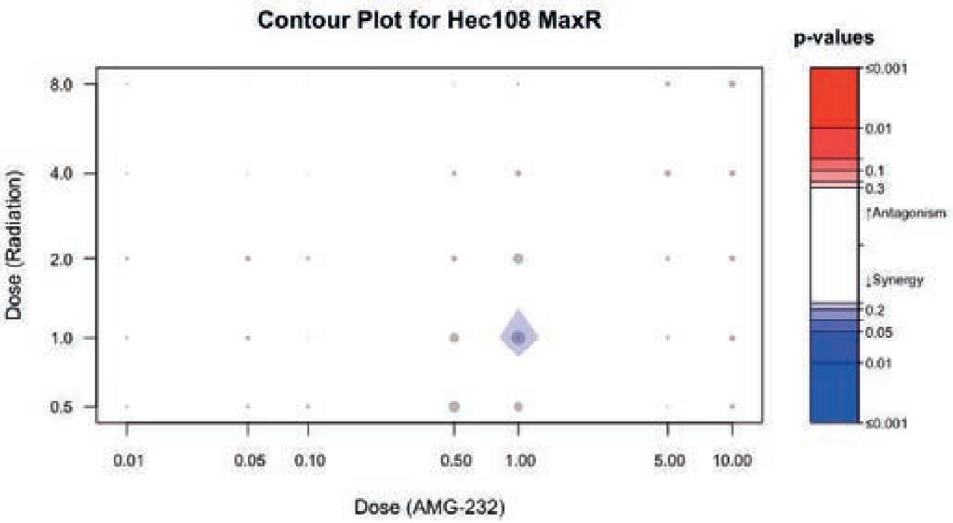

B.

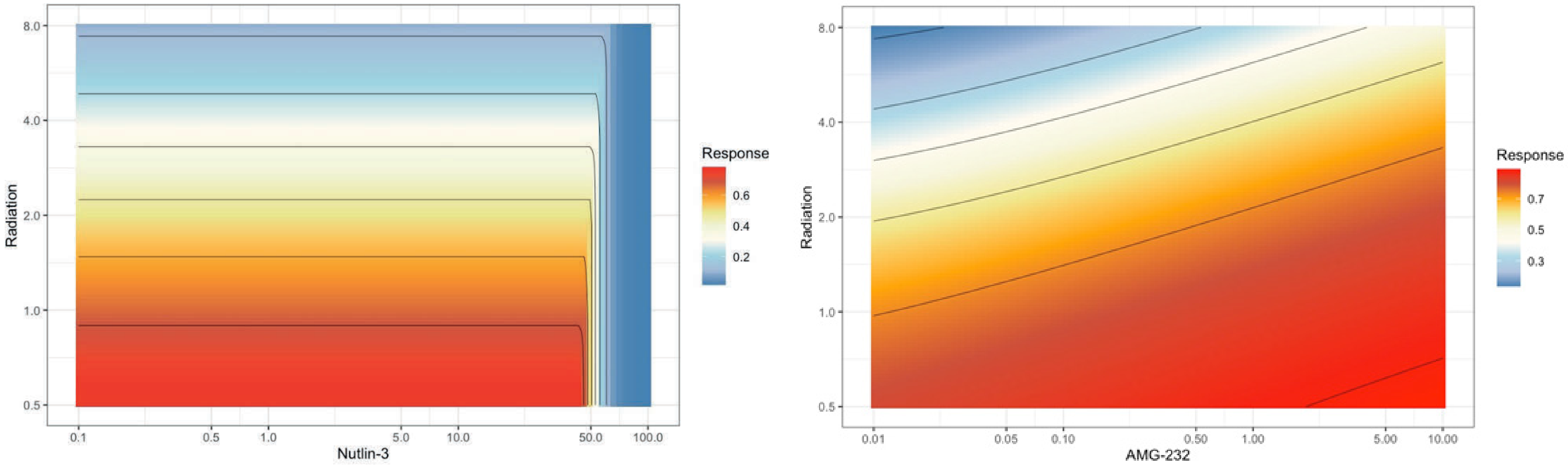

A) Contour plot of HEC108 and AMG-232 demonstrating weak synergy at 1nM and 1Gy dosing. **B)** Isobologram of Hec1B using radiation (y-axis) and drug concentration (x-axis). As noted there is no effect with the addition of Nutlin-3 to radiotherapy and a suggestion of an antagonistic effect at higher doses with AMG-232.

Supplemental Figure 5.

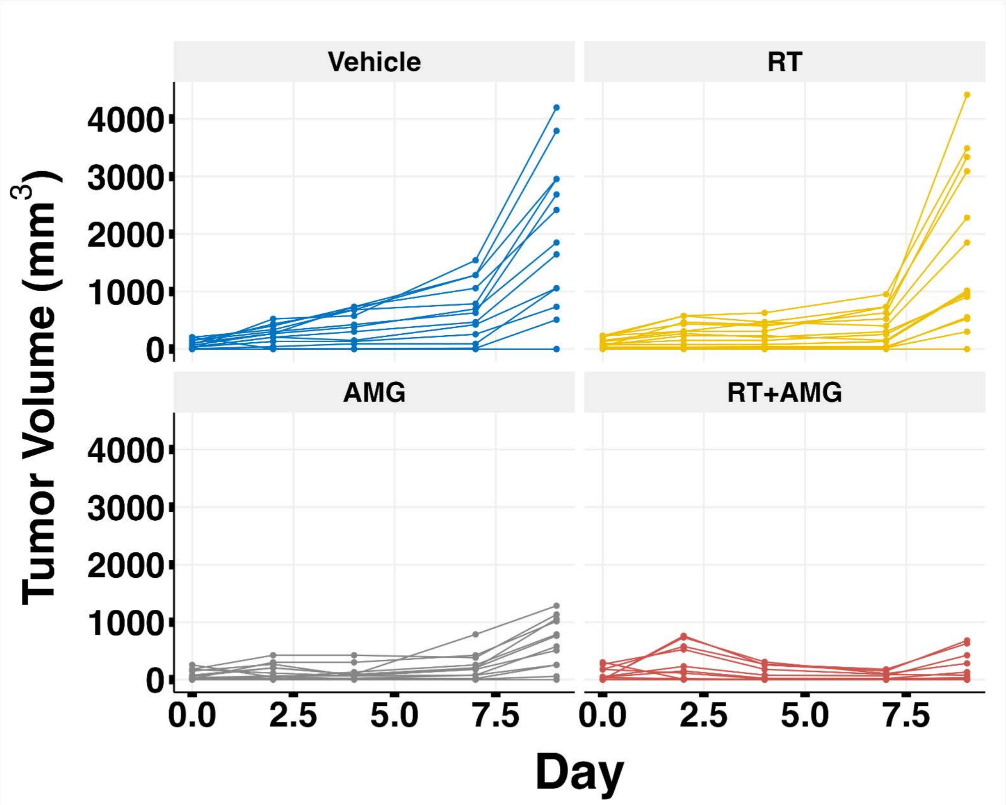

Individual xenograft tumor growth curves based on treatment group. Each line represents an individual tumor through the entire experiment. Each mouse had 2 tumors, one on each flank. We can observe that AMG-232 (bottom left) had a substantial impact on tumor size, while co-treatment with 1Gy of radiotherapy almost eliminated the tumors by day 7 post-treatment.

Supplemental Figure 6.

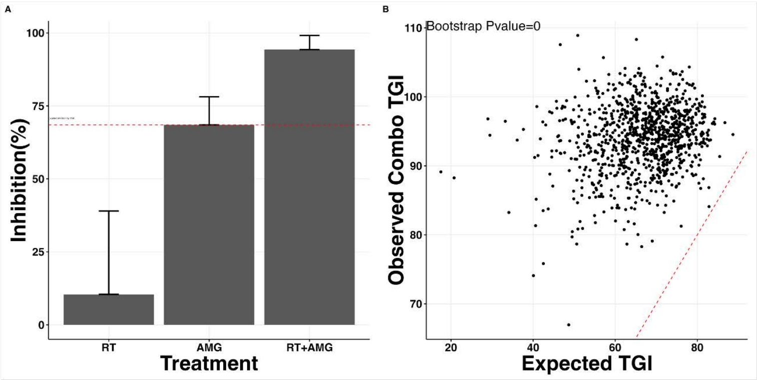

HSA

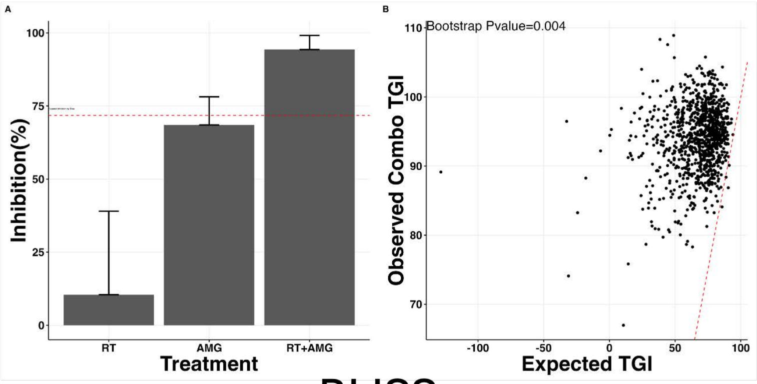

BLISS

In Vivo Highest Single Agent (top) and BLISS (bottom) synergy analyses for AMG-232 and Radiotherapy. As seen on the bar graphs (left) tumor growth inhibition (TGI) exceeded an additive effect under both models. Bootstrap analyses using both models (right) confirmed statistically significant synergy compared to expected TGI, as denoted by the dashed line.

Supplemental Table 1.

Term	Overlap	P-value	Adjusted P-value	Odds Ratio	Combined Score	Genes
**p53 Pathway**	**9/200**	**3.59E-06**	**1.26E-04**	**8.283096**	**103.8415782**	**CDKN1A;BTG2;SESN1;NINJ1;CCNG1;FAS;TM7SF3;TP53;BLCAP**
**Apoptosis**	**6/161**	**4.44E-04**	**0.007778**	**6.639215**	**51.24539677**	**CDKN1A;BTG2;CCND1;GADD45B;PLPPR4;FAS**
**TNF-alpha Signaling via NF-kB**	**6/200**	**0.001375**	**0.016047**	**5.294039**	**34.88214092**	**CDKN1A;BTG2;CCND1;GADD45B;NINJ1;IER2**
Mitotic Spindle	3/199	0.119967	0.422972	2.553139	5.414022257	RICTOR;KIFAP3;KIF15
Hypoxia	3/200	0.121286	0.422972	2.54005	5.358499671	CDKN1A;HOXB9;CCNG2
G2-M Checkpoint	3/200	0.121286	0.422972	2.54005	5.358499671	CCND1;MKI67;KIF15
Estrogen Response Early	3/200	0.121286	0.422972	2.54005	5.358499671	SOX3;CCND1;MYBL1
Myogenesis	3/200	0.121286	0.422972	2.54005	5.358499671	CDKN1A;GADD45B;SCHIP1
mTORC1 Signaling	3/200	0.121286	0.422972	2.54005	5.358499671	CDKN1A;BTG2;CCNG1
E2F Targets	3/200	0.121286	0.422972	2.54005	5.358499671	CDKN1A;MKI67;TP53

Hallmark pathway analyses using differential gene expression data 24 hours after radiotherapy exposure.

Supplemental Table 2.

Target	Target Sequence	F oligo (BsmBI site in red)	R oligo (BsmBI site in red)
NTC 1.1	GTATTACTGATATTGGTGGG	CACCGGTATTACTGATATTGGTGGG	AAACCCCACCAATATCAGTAATACC
NTC 3.1	TCAACCCCAGCGCACCGTTG	CACCGTCAACCCCAGCGCACCGTTG	AAACCAACGGTGCGCTGGGGTTGAC
TP53 5.1	TGAGGGCAGGGGAGTACTGT	CACCGTGAGGGCAGGGGAGTACTGT	AAACACAGTACTCCCCTGCCCTCAC
TP53 6.1	GTTGCAAACCAGACCTCAGG	CACCGGTTGCAAACCAGACCTCAGG	AAACCCTGAGGTCTGGTTTGCAACC

CRISPR/Cas9 guide-RNA sequences. A designed 5.1 targets exon 5 and 6.1 targets exon 6.

Supplemental Table 3.

**Name/mutation**	**F primer (mutated site underlined)**	**R primer (mutated site underlined)**
attL flanking sites	(L1) CGTTGTAAAACGACGGCCAGTC	(L2) GCCAGGAAACAGCTATGACCATG
TP53 R273H	AGCTTTGAGGTGCATGTTTGTGCCTGT	ACAGGCACAAACATGCACCTCAAAGCT
TP53 R273C	AGCTTTGAGGTGTGTGTTTGTGCCTGT	ACAGGCACAAACACACACCTCAAAGCT
TP53 R248Q	GGCGGCATGAACCAGAGGCCCATCCTC	GAGGATGGGCCTCTGGTTCATGCCGCC
TP53 R248W	GGCGGCATGAACTGGAGGCCCATCCTC	GAGGATGGGCCTCCAGTTCATGCCGCC
TP53 Y220C	GTGGTGGTGCCCTGTGAGCCGCCTGAG	CTCAGGCGGCTCACAGGGCACCACCAC

TP53 variant site-directed mutagenesis primer sequences with nucleotide substitutions.

Figure 1 - Panel **C** (JHUEM**1**)

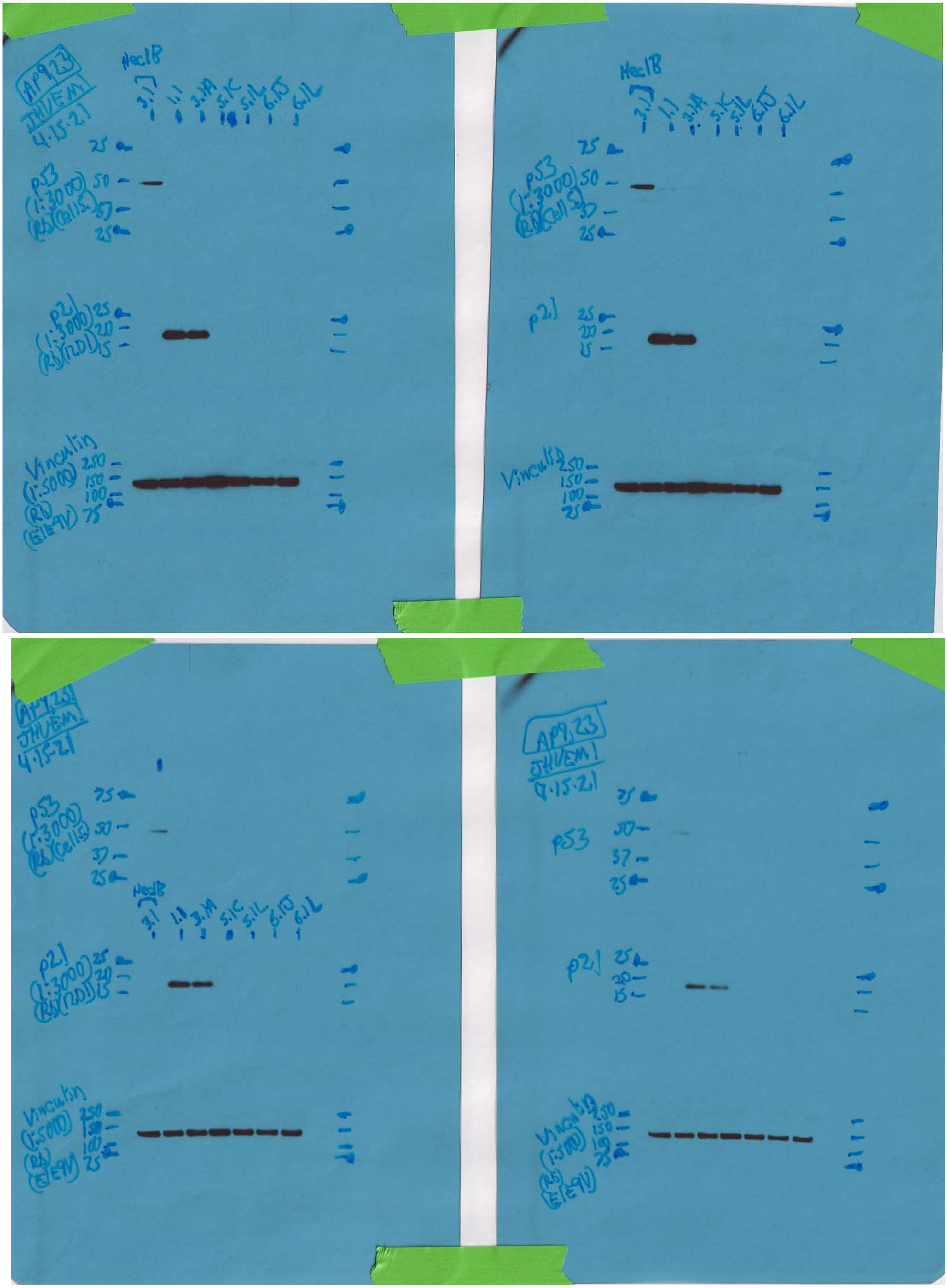

Figure 1 - Panel **C** (JHUEM**2**)

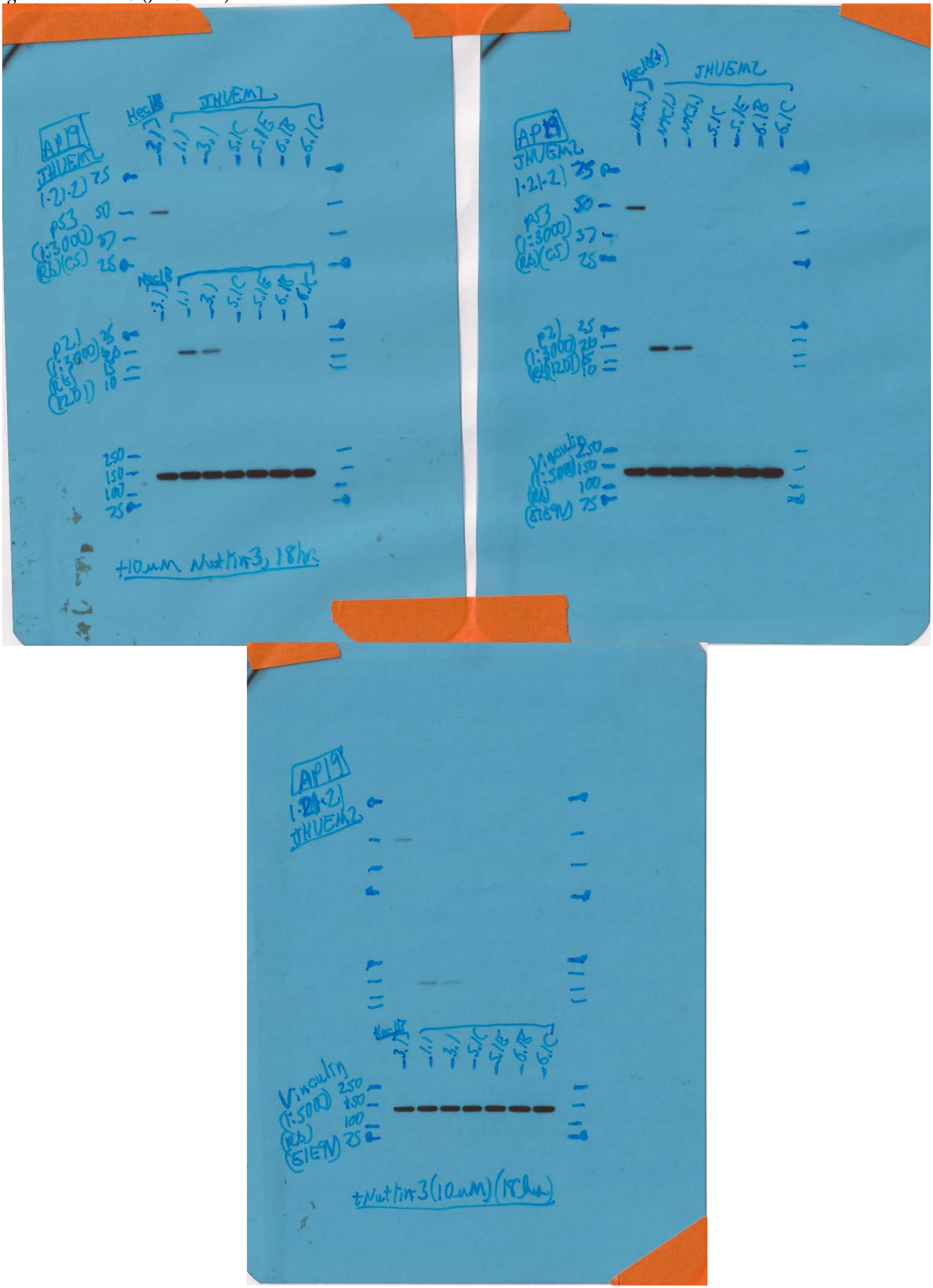

Figure 1 - Panel **D** (JHUEM2 - **5.1** TP53 KO Addback)

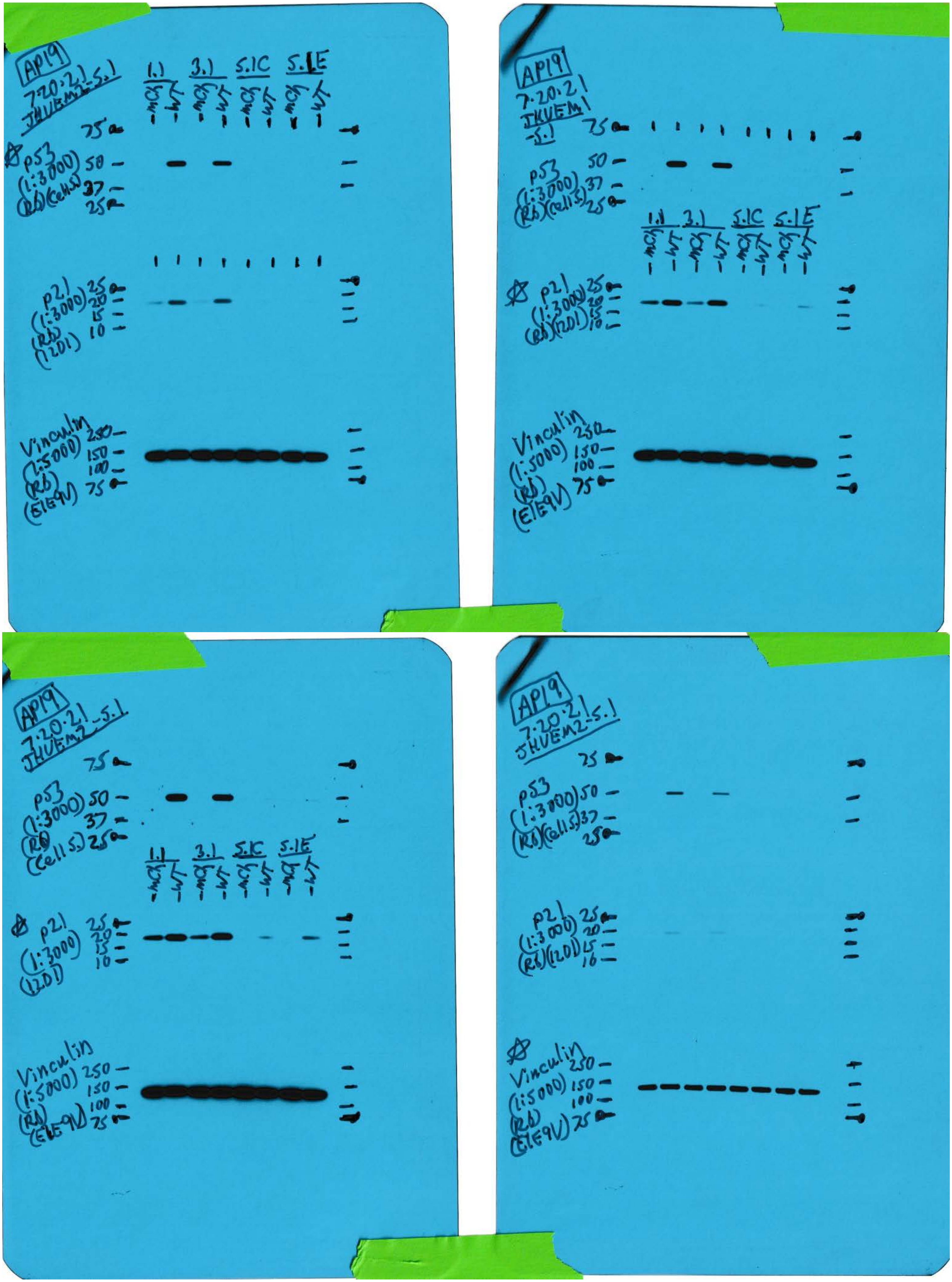

Figure 1 - Panel **D** (JHUEM2 - **6.1** TP53 KO Addback)

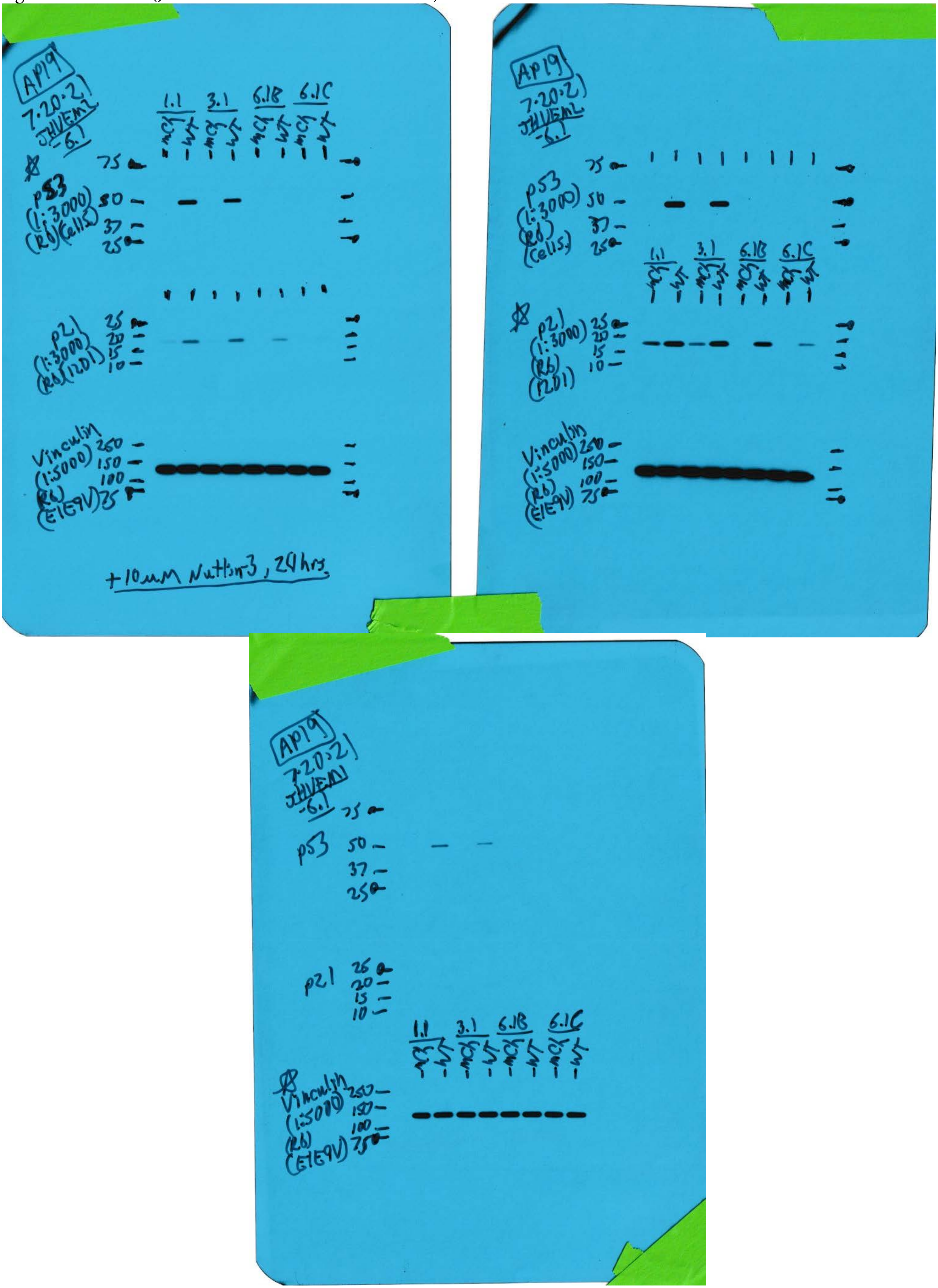

Figure 2 - Panel **A** (JHUEM2 + R273C Variant Dominant-Negative)

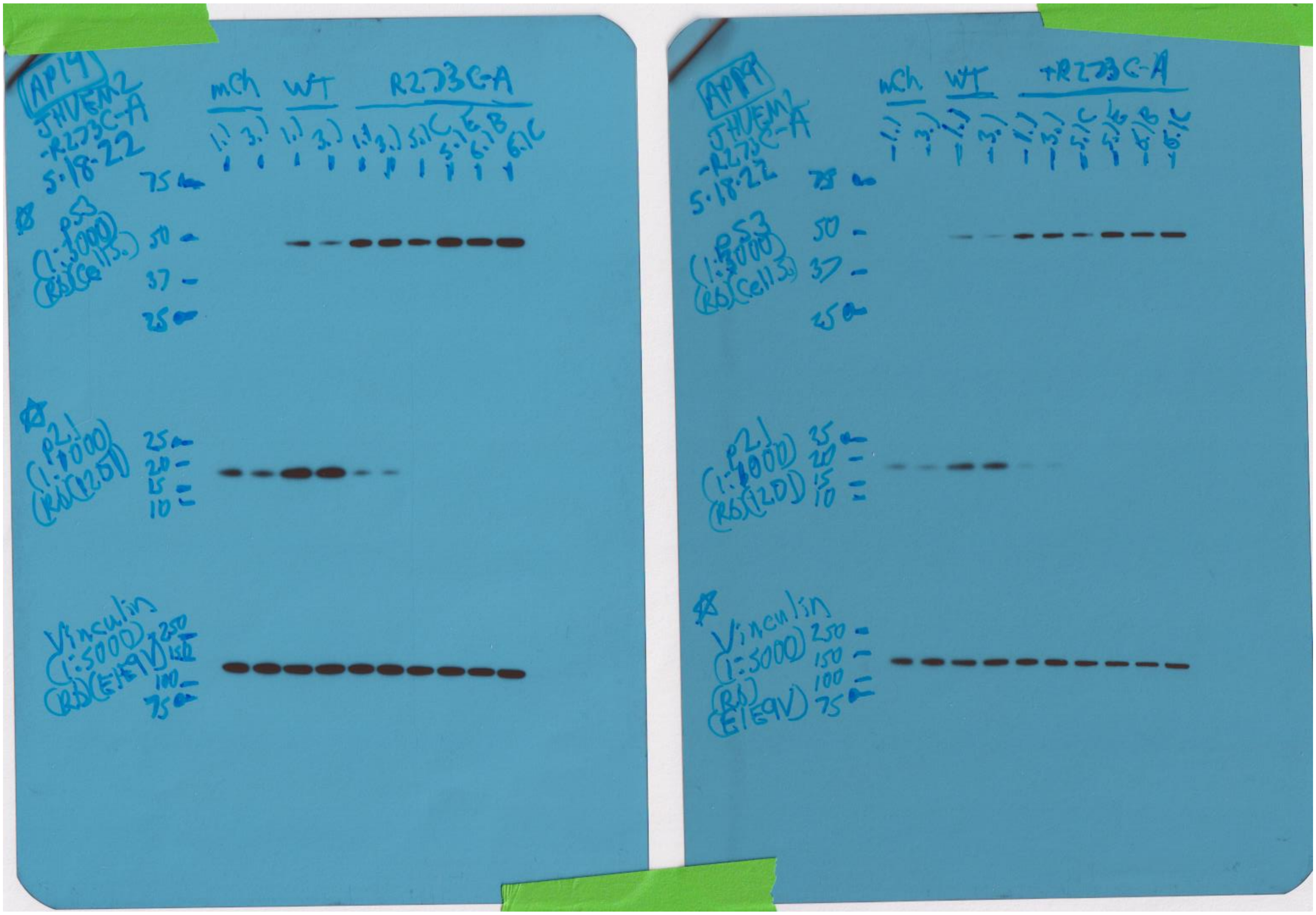

Figure 2 - Panel **C** (JHUEM2 + All TP53 Variants Dominant-Negative)

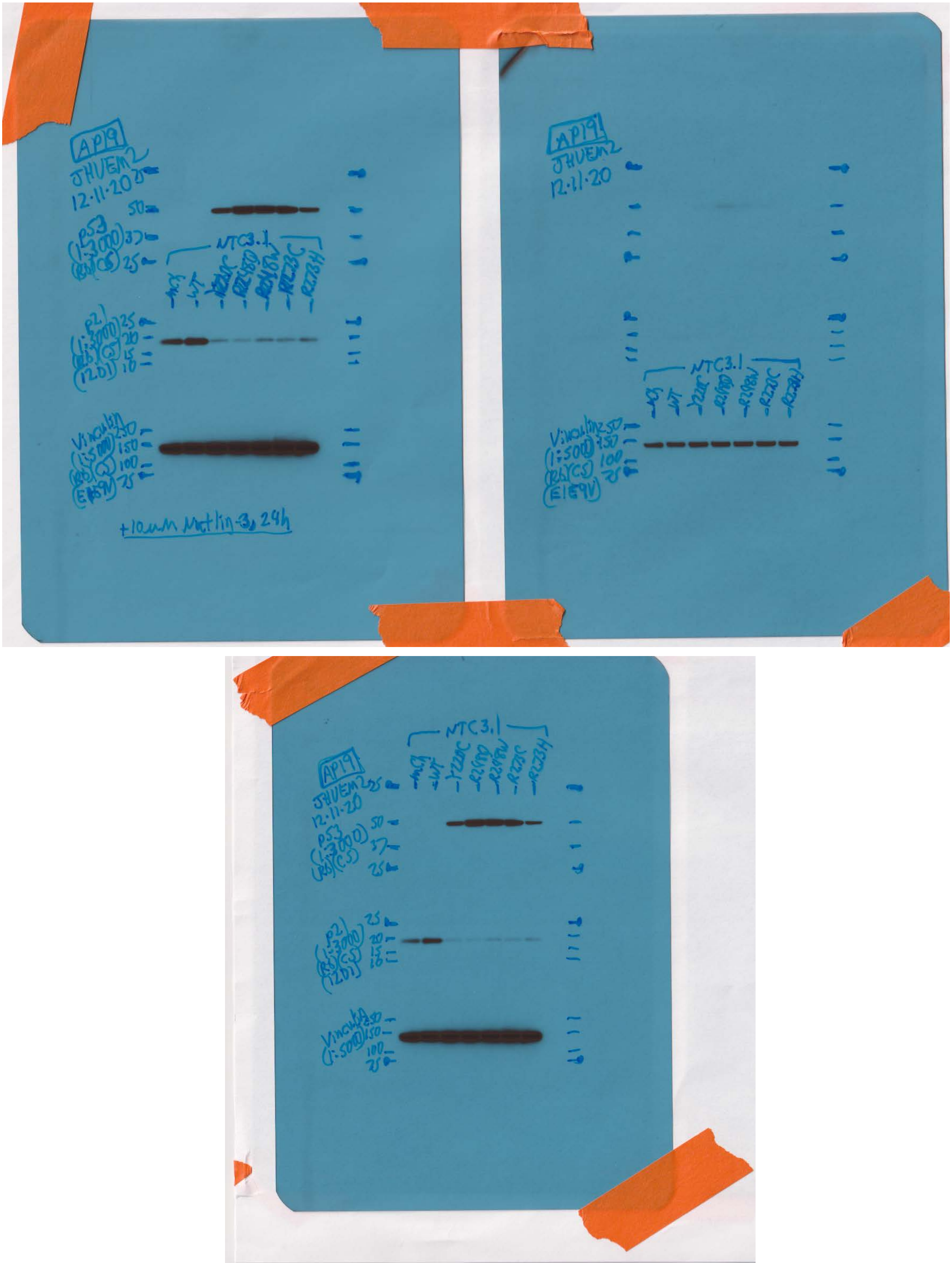

Figure **3** Panel **B** - (Nutlin-3 Response JHUEM2/Hec108/Hec1B)

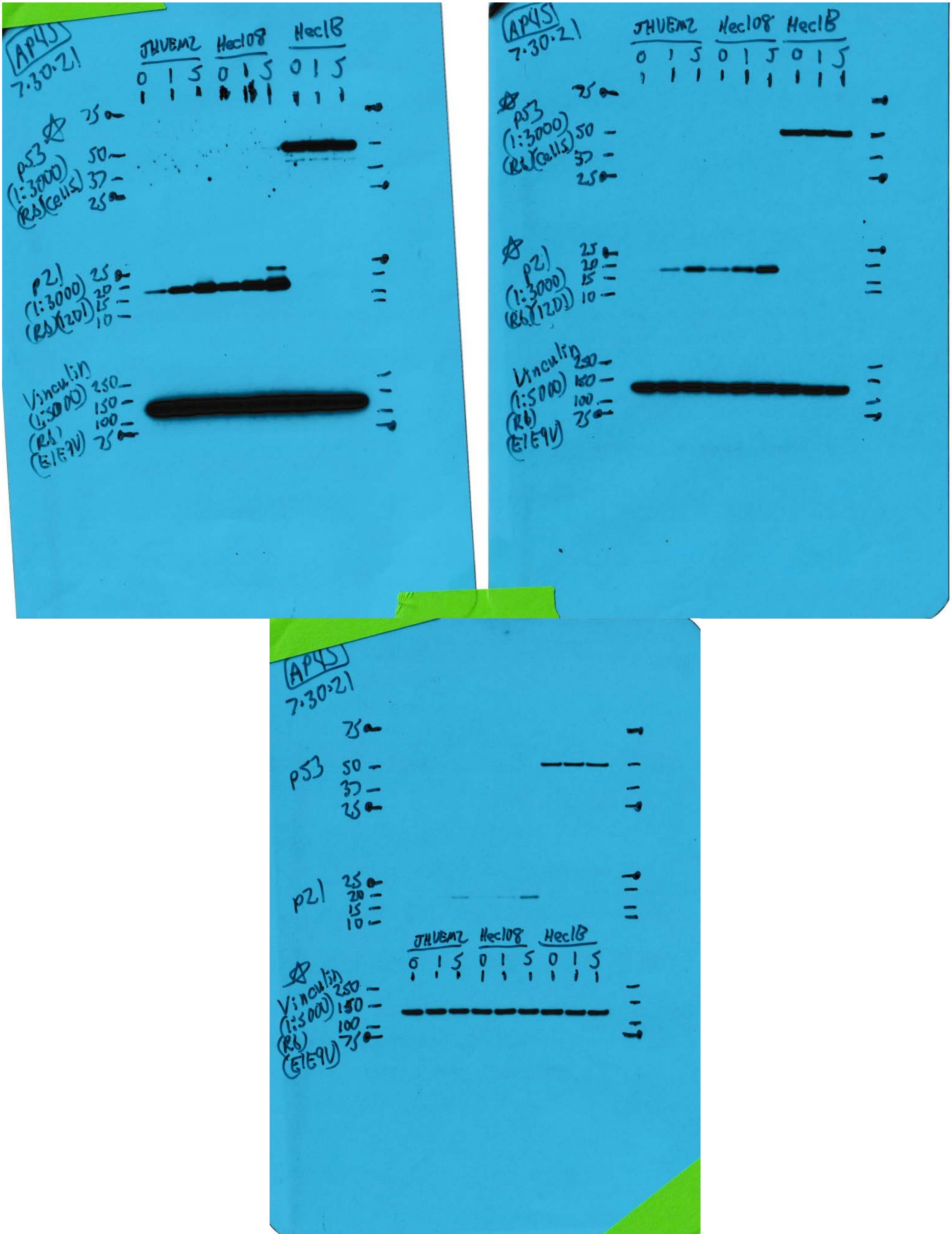

Figure **3** Panel **B**- (Nutlin-3 Response JHUEM2/Hec108 SubFigure)

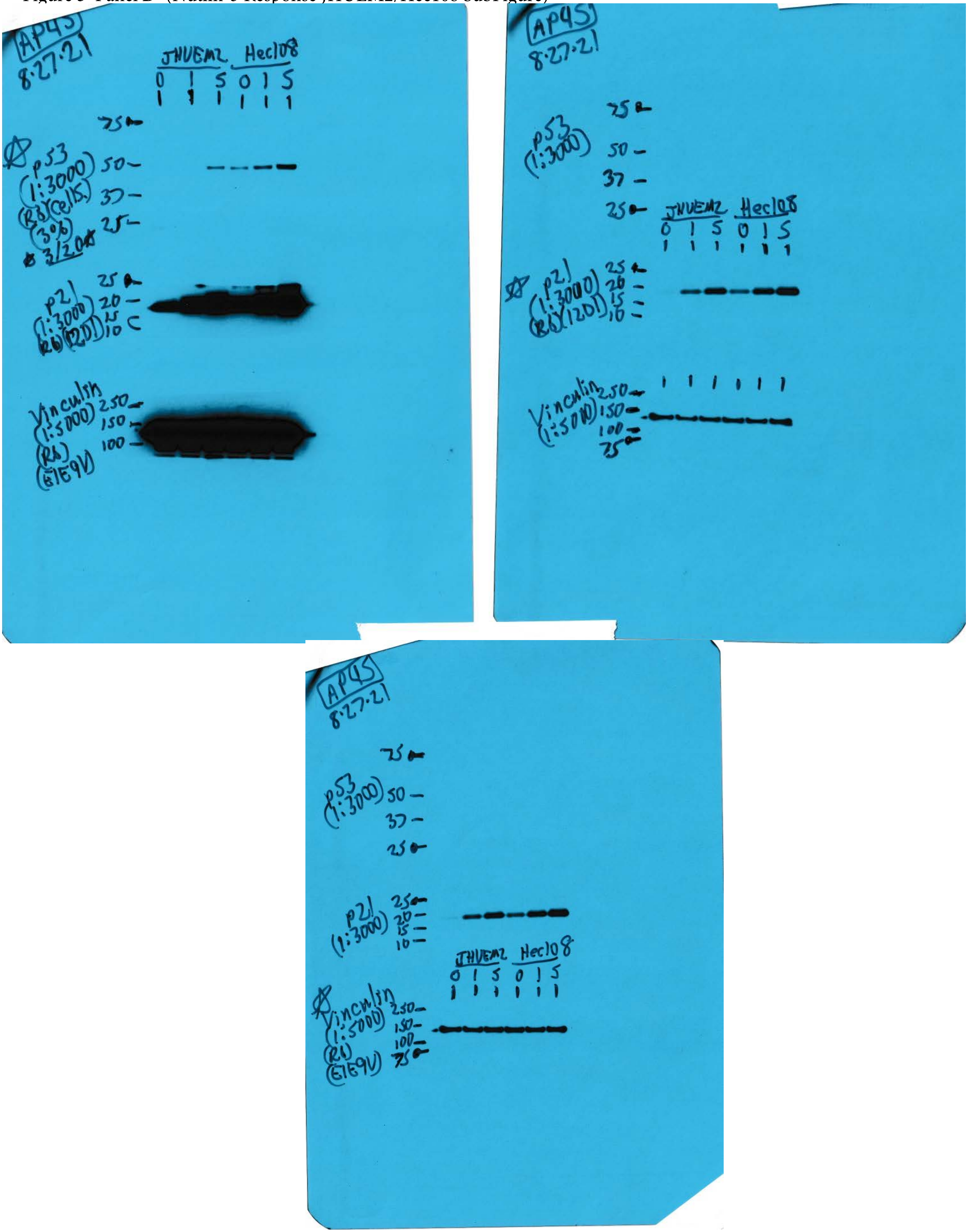

Figure 3 Panel **D**- (AMG-232 Response JHUEM2/Hec108/Hec1B)

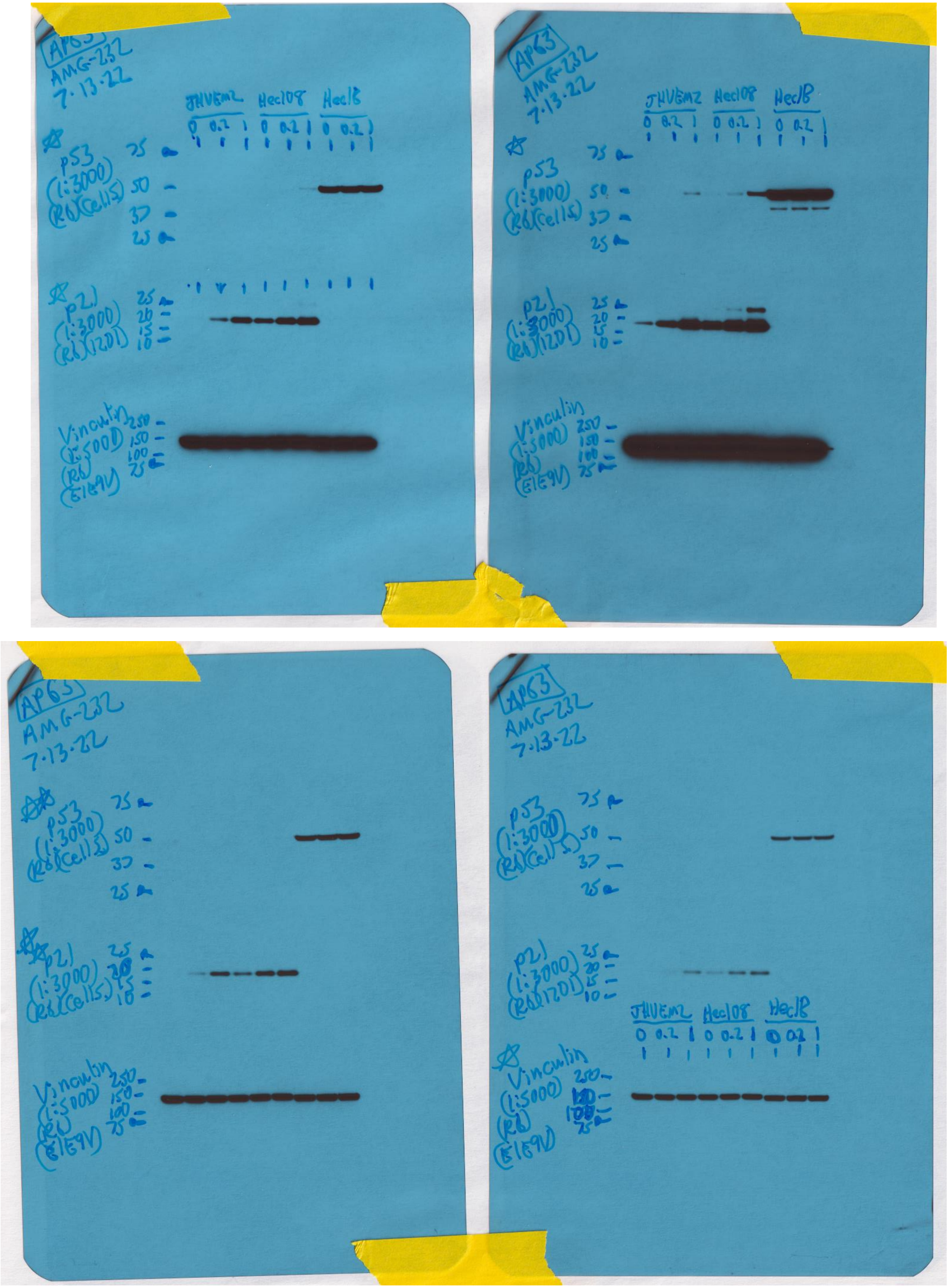

Supplementary Figure 2 - **Y220C**

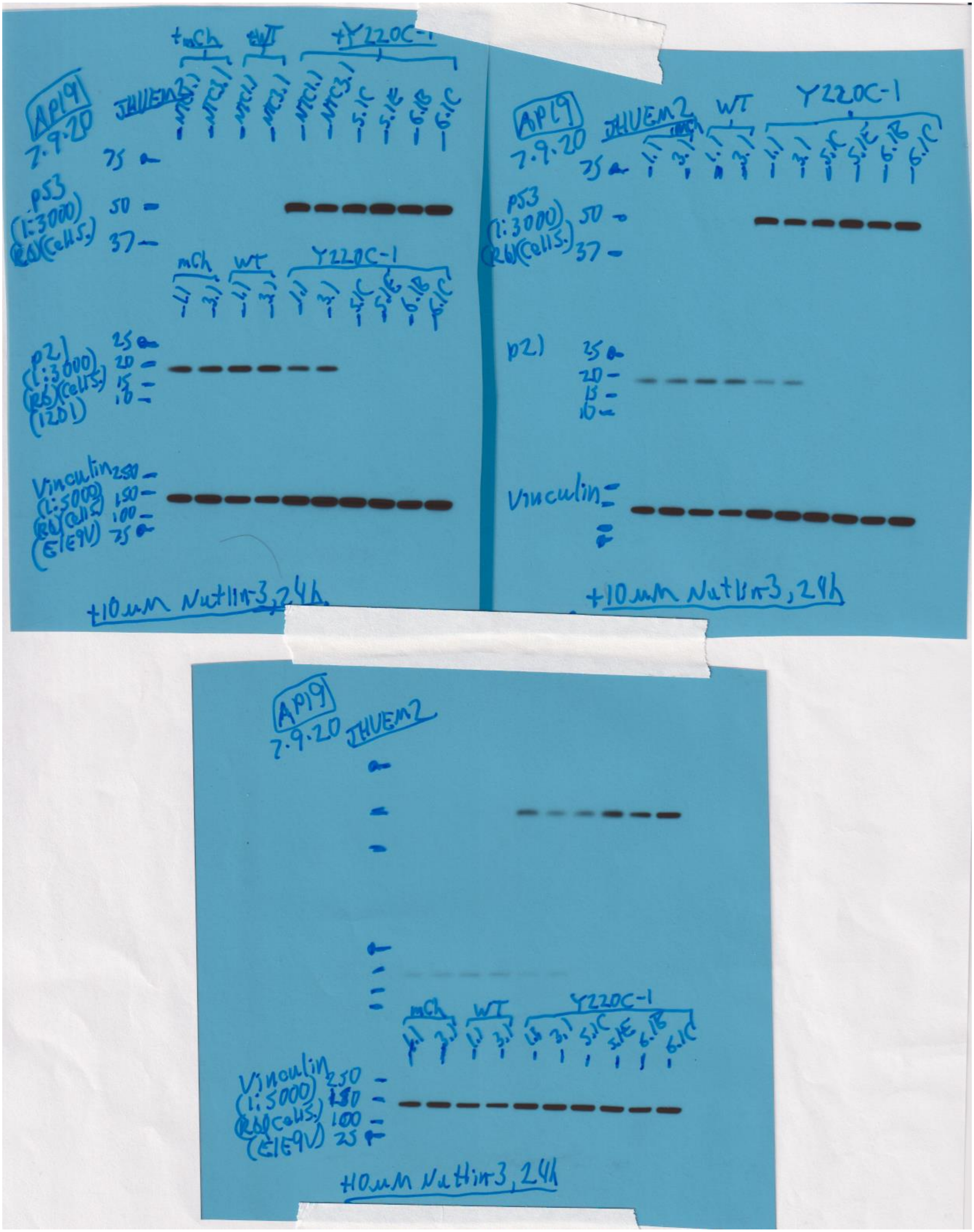

Supplementary Figure 2 - R248Q

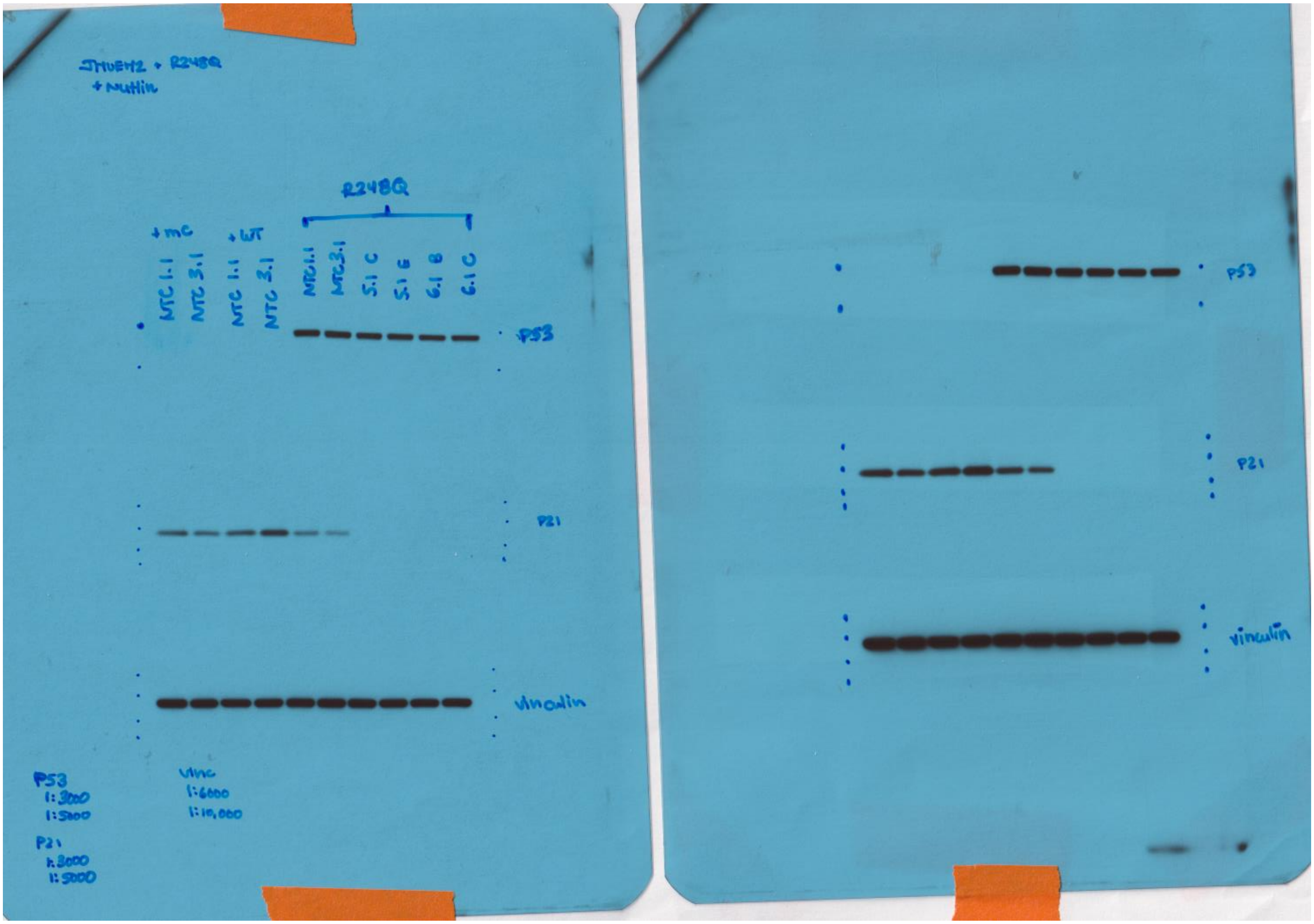

Supplementary Figure 2 - R248W

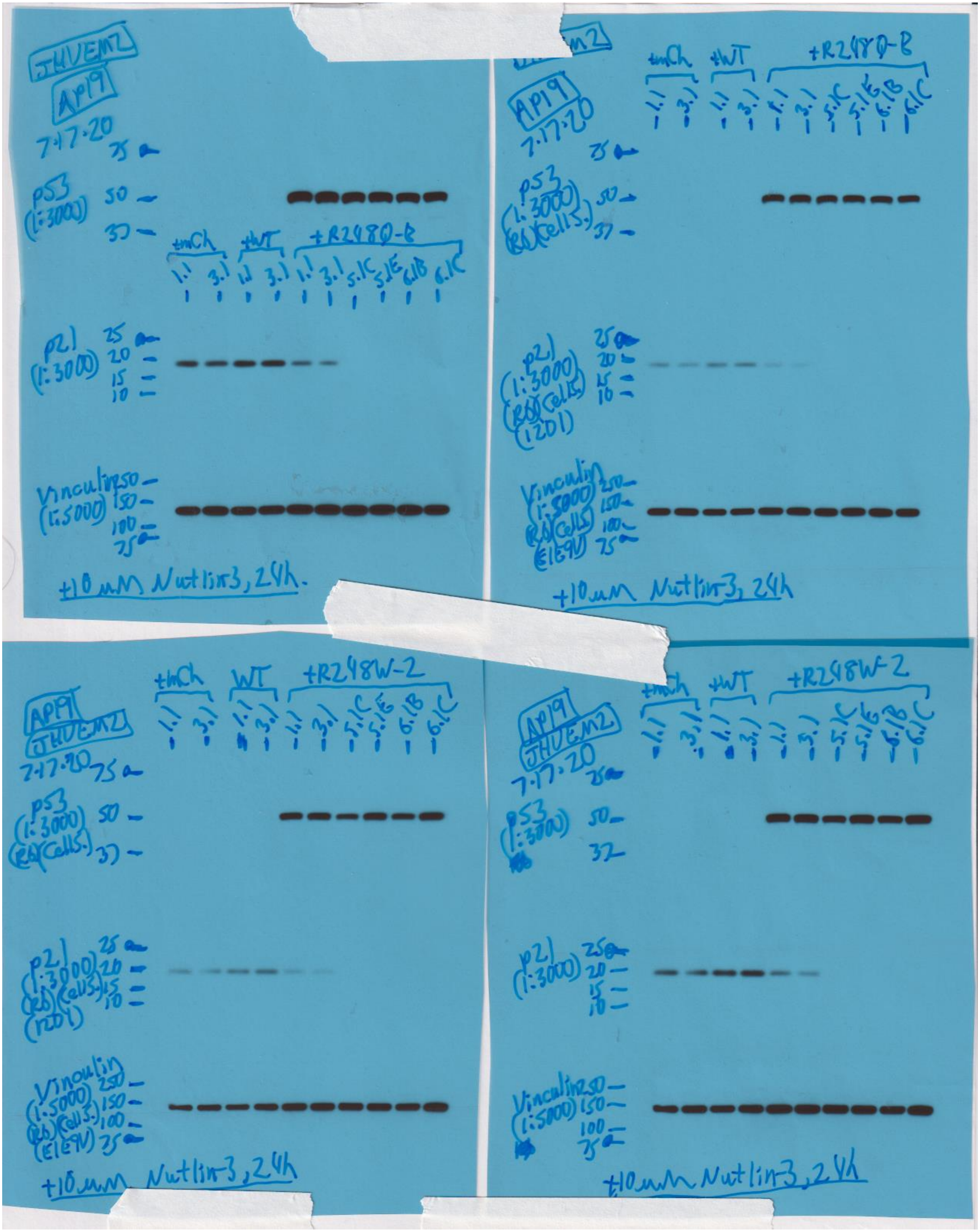

Supplementary Figure 2 - R273H

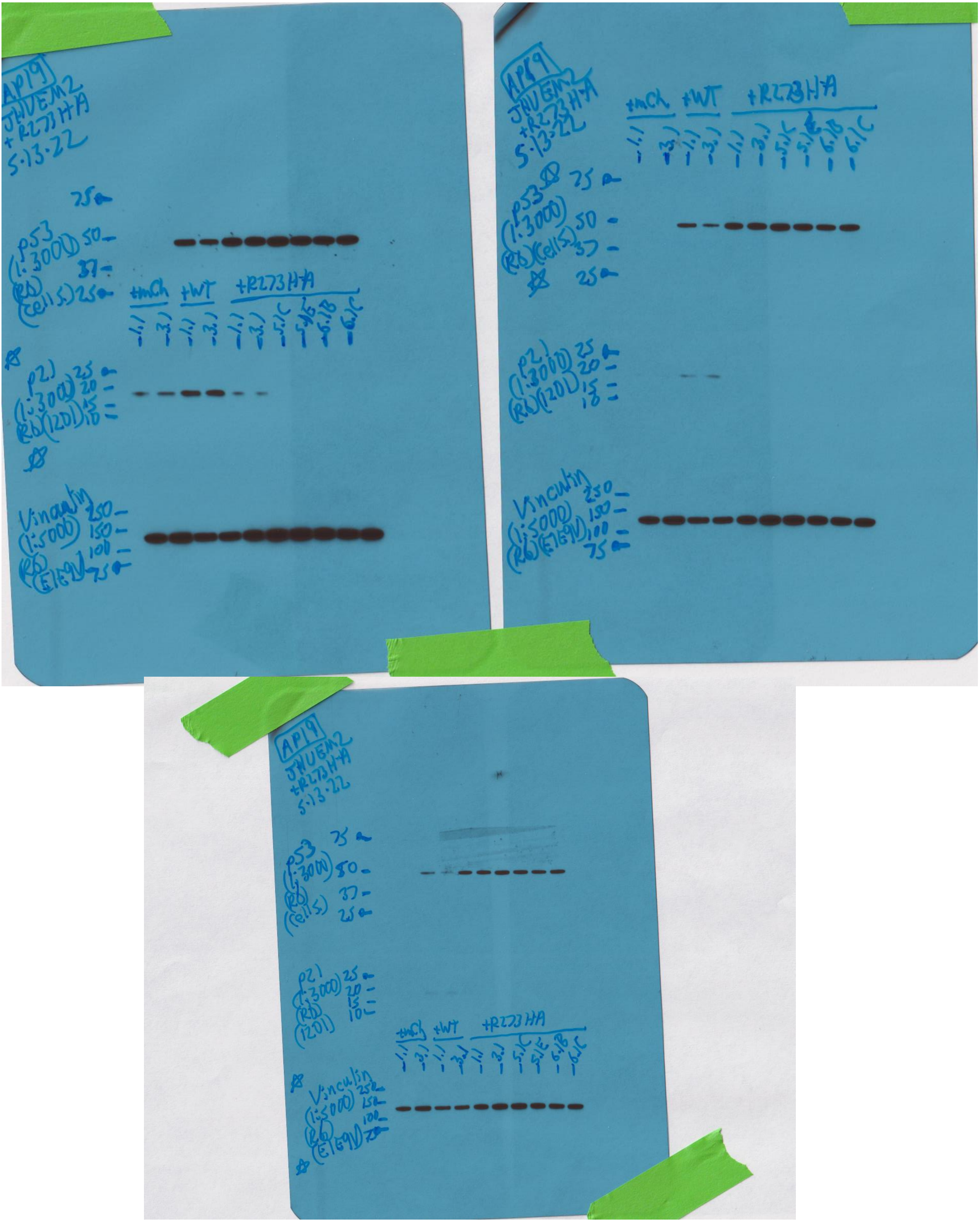

Supplement: Supplementary file 1 — Supplementary information [file 41698_2025_1063_MOESM1_ESM.pdf]
